# RS-SNP: a random-set method for genome-wide association studies

**DOI:** 10.1186/1471-2164-12-166

**Published:** 2011-03-30

**Authors:** Annarita D'Addabbo, Orazio Palmieri, Anna Latiano, Vito Annese, Sayan Mukherjee, Nicola Ancona

**Affiliations:** 1 Istituto di Studi sui Sistemi Intelligenti per l'Automazione - CNR, Via Amendola 122/D-I, 70126 Bari, Italy; 2 Ospedale "Casa Sollievo della Sofferenza" IRCCS, Laboratorio di Gastroenterologia, Foggia, Italy; 3 Departments of Statistical Science, Computer Science, Mathematics, Institute for Genome Sciences & Policy, Duke University, Durham, NC, USA

## Abstract

**Background:**

The typical objective of Genome-wide association (GWA) studies is to identify single-nucleotide polymorphisms (SNPs) and corresponding genes with the strongest evidence of association (the 'most-significant SNPs/genes' approach). Borrowing ideas from micro-array data analysis, we propose a new method, named RS-SNP, for detecting sets of genes enriched in SNPs moderately associated to the phenotype. RS-SNP assesses whether the number of significant SNPs, with p-value *P *≤ *α*, belonging to a given SNP set  is statistically significant. The rationale of proposed method is that two kinds of null hypotheses are taken into account simultaneously. In the first null model the genotype and the phenotype are assumed to be independent random variables and the null distribution is the probability of the number of significant SNPs in  greater than observed by chance. The second null model assumes the number of significant SNPs in  depends on the size of  and not on the identity of the SNPs in . Statistical significance is assessed using non-parametric permutation tests.

**Results:**

We applied RS-SNP to the Crohn's disease (CD) data set collected by the Wellcome Trust Case Control Consortium (WTCCC) and compared the results with GENGEN, an approach recently proposed in literature. The enrichment analysis using RS-SNP and the set of pathways contained in the MSigDB C2 CP pathway collection highlighted 86 pathways rich in SNPs weakly associated to CD. Of these, 47 were also indicated to be significant by GENGEN. Similar results were obtained using the MSigDB C5 pathway collection. Many of the pathways found to be enriched by RS-SNP have a well-known connection to CD and often with inflammatory diseases.

**Conclusions:**

The proposed method is a valuable alternative to other techniques for enrichment analysis of SNP sets. It is well founded from a theoretical and statistical perspective. Moreover, the experimental comparison with GENGEN highlights that it is more robust with respect to false positive findings.

## Background

The objective of genome-wide association studies (GWAS) is to identify genetic variants, a subset of single nucleotide polymorphisms (SNPs), associated with the onset and progression of complex disease phenotypes at a genome-wide scale [1]. Although GWAS have identified numerous loci with strong association with common polygenic diseases [2], these studies have some limitations. The main source of these limitations is that SNPs are analyzed independently, requiring large sample sizes and strong associations to detect an effect. It is also very difficult using this approach to identify and incorporate weakly associated SNPs into the analysis. For polygenic diseases focusing the analysis on only the most significant SNPs is particularly problematic as no particular gene may have a large effect [1] but genic regions weakly associated to the phenotype are important when susceptibility is conferred by a large number of loci, each with a small effect on risk for the disease.

Recently a new trend is emerging in genetics and computational biology in which groups of genes are analyzed simultaneously for association with a phenotype or disease [3-5]. The gene sets can be derived from different sources, for example the sets of genes representing biological pathways or sets of genes proximal to each other. By borrowing strength across the gene set, there is potential for increased statistical power. In addition, in comparing study results on the same disease from different labs, gene set approaches may be more reproducible than from individual gene studies [6]. In the analysis of gene expression data, this approach is effective at targeting groups of genes whose constituents show subtle but coordinated expression changes, this may not be detected by individual gene analysis. The approach has been quite successful in deriving new information from expression data [7], and tools developed for gene set enrichment analysis of gene expression data abound [8].

The same principle has been recently applied in GWAS for assessing association of sets of SNPs and phenotypes [9-12], and many of the proposed approaches can be considered as extensions to SNP analysis of a method, Gene Set Enrichment Analysis (GSEA) [3], designed for micro-array data analysis. Examples are the methods proposed by Wang et al. [13] and Holden et al. [14]. The basic idea for both these methodologies is to assign for each gene a correlation statistic of the gene with the phenotype analogous to the correlation of the phenotype with expression level of a gene in GSEA. The method proposed by Wang et al. [13] considers a correlation score based on the most significant SNP of all the SNPs mapped to a given gene and uses a Kolmogorov-Smirnov like statistics for assessing enrichment. The approach implemented by Holden et al. [14] computes the correlation statistic based on the association of all the SNPs mapped to a gene and evaluates the enrichment score of a gene set by comparing the SNPs in the gene set with the list of the most associated SNPs in the data set.

In this paper we describe a new methodology that assesses the association of gene sets to a trait by including simultaneously strong association signals as well as SNPs moderately associated to the phenotype. The approach belongs to the general class of Random Set methods [6,15] designed to assess enrichment of gene sets in microarray gene expression data analysis. Our method, named RS-SNP, assesses whether the number of significant SNPs (p-value *P *≤ *α*,) belonging to a given gene (SNP) set is statistically significant. The null model we specify assumes that the genotype and the phenotype are independent and the number of significant SNPs does not depend on the identity of the SNP set, but only on the size of the gene (SNP) set. We use non-parametric permutation procedures [16] to test against the null. This preserves the linkage disequilibrium (LD) structure for SNPs in a given chromosomal region. The performance of RS-SNP on the Crohn's disease (CD) data set collected by the Wellcome Trust Case Control Consortium (WTCCC) [2] has been evaluated and compared to the method proposed by Wang et al. [13], indicated as GENGEN.

## Implementation

### Defining a SNP set

Before introducing a detailed description of the method used to perform SNP set analysis, it is important to clarify how a SNP set can be defined.

The first step in defining a SNP set is mapping SNPs to genes. SNPs may fall within coding regions of genes, non-coding regions of genes, or in the inter-genic regions between genes. Each SNP *V_i _*in a GWA study, with *i *= 1,..., *n*, is associated to a gene *G_j_*, where *j *indexes the total *M *genes, if the gene contains the SNP or is the closest gene to the SNP. In cases where a SNP is located within shared regions of two overlapping genes, it is mapped into both genes. SNPs that are a fixed number of kilo-bases (kb) away from any gene are not considered. In [13] SNPs are associated to a given gene if they are within 500 kb. The selection of 500 kb is due to most enhancers and repressors being <500 kb away from genes and most LD blocks being <500 kb.

The second step is mapping genes to pathways. The pathways are pre-defined lists of genes based on a *a priori *biological knowledge, for example genes which are co-expressed in a particular cellular mechanism or function [17-19]. We use the Molecular Signatures Database (MSigDB) [3] as a source of gene pathways.

### Random set methods

Random Set (RS) scoring methods were primary introduced by Efron and Tibshirani [6] to study the enrichment signal in gene sets analysis by using gene expression data. The methods they proposed are more widely applicable.

The main idea pointed out by RS methods is that any method for assessing gene sets should compare a given gene set score not only to scores from permutations of the sample labels, but also taking into account scores from sets formed by random selections of genes.

In fact, any approach to gene set analysis begins with the computation of some enrichment score , for each gene set , and computes its significance by comparison with permutation values . Efron and Tibshirani in [6] argue that a second kind of comparison operation, called "row randomization", is also needed to avoid bias in the determination of significance.

In order to better clarify RS positions let us consider a simplified statement of the gene set problem, proposed by Efron and Tibshirani but adapted to the SNP data framework.

Let **X **indicate an *n *× *ℓ *matrix of genotypic observations, where *n *is the total number of SNPs and *ℓ *is the total number of samples, with the first *ℓ*_1 _columns of **X **representing healthy control samples and the remaining *ℓ*_2 _are case samples, *ℓ*_1 _+ *ℓ*_2 _= *ℓ*. A statistic *D_i_*, *i *= 1,..., *n *is computed for each marker. Consider a single gene set  with *m *genes and the hypothesis that  is enriched. Testing this hypothesis is equivalent to asking if the *m *D-values have large magnitude (positive or negative), with large to be defined. The basic idea underlying enrichment, as nicely stated by Subramanian [3], is that a biologically related set of genes can be detected from the general effect of its D constituent values whether or not the individual genes are significantly non-zero. Let  indicate the set of *m *D-values in  and ( defines an enrichment test statistic, with larger value of ES indicating greater enrichment. Testing  for enrichment requires a distribution under the null hypothesis for ES. The following are two quite different models for what the null hypothesis might mean:

• **Permutation Model**. Let **X***_S _*be the *m *× *ℓ *submatrix of **X **corresponding to . The null hypothesis  is that the *ℓ *columns of  are independent and identically distributed *m*-vectors (i.i.d.). The null density of ES, , is obtained by column permutations.

• **Randomization Model**. The null hypothesis  is that  has been chosen by random selection of *m *SNPs from the full set of *n *SNPs. In this case the null density of ES, say , can be obtained by row randomization: sets  of *m *rows of the data matrix **X **are drawn at random, giving randomized values . These randomized values are computed and used to construct an empirical estimate of .

The randomization of the markers and the permutation of the labels can be combined into a method that is called "Restandardization". Restandardization can be thought as a method for correcting the permutation values of ES to take into account the overall null distribution of ES in the randomization model. The restandardized enrichment score (RES) used is defined as:(1)

where (*μ*^†^, *σ*^†^) are the mean and standard deviation of *ES*^† ^and (μ*, σ*) are the corresponding quantities based on label permutations. Two nested permutation procedures are needed in this case which is computationally intensive. Fortunately, the RS method has an appealing feature: for certain choices of the summary statistic  the restandardized score can be easily computed by analytically calculating the gene-wise means and standard deviations, without having to draw random set of genes. As a result evaluation of statistical significance requires only label permutations [6,15].

### Random Set method for SNP data: RS-SNP

RS-SNP is designed for genome-wide SNP data with binary categorical phenotypes, for example cases and healthy controls.

The first step in the method is computing a correlation or association statistic *D_i _*for each SNP *V_i_*, *i *= 1,...., *n*. The association of a SNP with a trait can be assessed by considering five different genetic models [20]: general, dominant, recessive, multiplicative risk and additive risk model. The first three models use a *χ*^2 ^test (or Fisher's exact test) on genotype entries to compute association. The multiplicative risk model uses a *χ*^2 ^test or Fisher's exact test on allelic entries to compute association. The additive risk model uses a Cochran-Armitage test for trend [21] to associate a SNP to disease risk of association.

After computing the single SNP associations, RS-SNP computes the enrichment of these associations in a predefined gene set . The mapping of each SNP *V_i _*to genes is discussed above. The relevant components of the method include:

• *n *= the number of genotyped SNPs;

• *d *= the number of SNPs with p-value *P *less than or equal to a given threshold *α*;

• *m *= the number of SNPs in ;

• *y *= the number of SNPs belonging to  with p-value *P *≤ *α*.

RS-SNP assesses whether the number *y *of SNPs associated to the phenotype and belonging to  is compatible with chance or indicates over-representation of associated SNPs in gene set . Assessing the statistical significance of *y *requires the distribution of *y *under two null hypotheses, as previously stated [6]. The first null hypothesis considered is the hypothesis in which there is no association between genotype and phenotype (). In particular, the method assesses the probability of observing values of *y *greater than the observed ones when genotype and phenotype are independent random variables. In addition, a second cause of randomness for *y *comes from . Knowing that *d *of *n *SNPs have p-value *P ≤ *α and that *y *of them fall in a gene set  with size *m*, how many SNPs fall in a set composed of *m *SNPs drawn randomly from the *n *SNPs available? To take into account this source of randomness, the probability of observing values of *y *greater than the ones observed in the actual experimental conditions has to be assessed under the hypothesis () that the *m *loci in the gene set  have been chosen randomly from the full set of *n *SNPs. Note that this problem is easy to solve since under this model the distribution for *y *is hypergeometric *Hyp*(*m*, *d*, *n*) with mean  and variance . To assess the statistical significance of *y *under the two null hypotheses simultaneously, the following procedure is carried out.

(1) Permute the labels of the samples Π times. For each permutation *π *= 1, ..., Π:

(i) Compute the number of significant SNPs .

(ii) Compute the number of significant SNPs belonging to , .

(iii) Compute the mean  and variance  under the hypergeometric distribution .

(iv) From the above , , and  compute .

(2) Compute the p-value

where *I *is the indicator function.

Since several gene sets are considered in the analysis, the false-discovery rate (FDR) and the family-wise error rate (FWER) are computed as proposed by Wang et al. [13] in order to control multiple hypothesis testing.

FDR, i.e. the fraction of expected false-positive findings, is calculated as:

where *T *is the total number of gene sets. The FWER is evaluated as the fraction of all permutations whose highest standardized enrichment score in all gene sets is higher than the *RES*() for a given gene set:

## Results and Discussion

### Experimental data set

#### WTCCC data set

The data set provided by WTCCC is composed of 2005 Crohn's Disease (CD) patients and 3004 healthy controls (HC). The control individuals came from two sources: 1504 individuals from the 1958 British Birth Cohort (58 C) and 1500 individuals selected from blood donors recruited as part of the WTCCC project (UK Blood Services (UKBS) controls). All 5009 samples were genotyped with the GeneChip 500 K Mapping Array set (Affymetrix chip), which comprises 500,568 SNPs. The quality control analysis was carried out following the details specified by WTCCC [2]. In particular, 257 CD and 66 HC subjects were excluded from the study, reducing the number of CD to 1748 and the number of HC to 2938 subjects. Moreover, markers were excluded with the following criteria:

• SNPs with Hardy-Weinberg exact p-value *P *< 5.7 × 10^-7 ^in the combined set of 2938 controls;

• SNPs with p-value *P *< 5.7 × 10^-7 ^for either a one or two-degree of freedom test of association between the two control groups;

• SNPs with a *MAF *< 1%.

In total, *n *= 414, 483 SNPs in autosomal chromosomes passed the quality control filters. More detailed information on WTCCC data set are in [2].

#### SNP set construction

Two different collections of gene sets were used, that can be downloaded from the MSigDB website http://www.broad.mit.edu/gsea/msigdb/index.jsp:

• MSigDB C2 CP collection, composed of pathways collected from various sources such as online databases, biomedical literature in PubMed, and knowledge of domain experts. In particular, the canonical pathways (CP) collection consists of 639 gene sets;

• MSigDB C5 collection, composed of 1454 gene sets derived from Gene Ontology (GO). This collection is composed of 825 GO biological processes, 233 GO cellular components and 396 GO molecular functions. We have considered only those GO terms associated with a specific reference that describes the work or analysis upon which the association between a specific GO term and gene product is based. Each annotation includes an evidence code to indicate how the annotation to a particular term is supported http://www.geneontology.org/GO.evidence.shtml. Only associations with the following evidence codes are included in MSigDB gene sets: IDA IPI, IMP IGI, IEP ISS, TAS. Moreover, GO gene sets for very broad categories, such as Biological Process, have been omitted from MSigDB. GO gene sets with fewer than 10 genes have also been omitted. Gene sets with the same members have been resolved based on the GO tree structure: if a parent term has only one child term and their gene sets have the same members, the child gene set is omitted; if the gene sets of sibling terms have the same members, the sibling gene sets are omitted.

The mapping of SNPs to genes has been carried out by using the Affymetrix annotation files Mapping250 K Nsp Annotations and Mapping250 K Sty Annotations, CSV format, version 26. In this study, SNPs were assigned to a given gene if they are within 5 kb from it.

### Experimental results of RS-SNP and GENGEN

#### Results on MSigDB C2 CP collection

The association of each SNP to CD was assessed by using the Cochran-Armitage trend test with 1 degree of freedom [21]. A significance threshold *α *= 0.01 was used and *d *= 6803 signals with p-value *P *≤ *α *were found.

Statistical significance and adjustment for multiple hypothesis testing were determined by a permutation-based procedure with Π = 10,000 random permutations of the phenotypic status of the subjects. The FDR and FWER were also computed.

The enrichment analysis highlighted 86 pathways (p-value *P *≤ 0.05) enriched in SNPs weakly associated to the trait. The enrichment analysis, performed by GENGEN on C2 CP collection, highlighted 115 pathways (p-value *P *≤ 0.05) enriched in SNPs weakly associated to the trait. Intersecting the two lists of gene sets found to be significant by RS-SNP and GENGEN resulted in 47 pathways.

Detailed tables, concerning the list of significant pathways in MSigDB C2 collection obtained by RS-SNP and GENGEN methods, are reported in the additional file 1.

#### Results on MSigDB C5 collection

The association of each SNP to CD was computed using the same methodology as above. Statistical significance and adjustment for multiple hypothesis testing was also estimated using the same procedure as stated above with Π = 10,000 random permutations of the phenotypic status of the subjects.

The enrichment analysis performed by RS-SNP on the MSigDB C5 collection highlighted 196 pathways (p-value *P *≤ 0.05) enriched in SNPs weakly associated to the trait. The enrichment analysis performed by GENGEN on MSigDB C5 collection highlighted 256 *pathways *(p-value *P *≤ 0.05) enriched in SNPs weakly associated to the trait. Intersecting the lists of gene sets resulted in 89 *pathways*.

Detailed tables, concerning the list of significant pathways in MSigDB C5 collection obtained by RS-SNP and GENGEN methods, are reported in the additional file 2.

#### Computational complexity evaluation

To evaluate and compare the computational cost of RS-SNP and GENGEN we used a computer equipped with two quadcore 2.67 GHz processors, 24 Gbyte of RAM, working under Linux OS. The first step, common to both the algorithms, was to assess the association between each SNP and the phenotype. The computation of the additive trend test statistics on the whole set of markers available in the WTCCC data required 18 sec for the actual phenotypic status of the samples and 50 min for random permutations of their phenotypic status. The second step was to assess the statistical significance of the enrichment score, under both the null and alternative hypotheses, for each of the 639 gene sets of the considered C2 CP collection. This step required 29 min for RS-SNP and 50 min for GENGEN. These computational costs indicate that the algorithmic complexity of both approaches is comparable.

## Discussion

We conclude with a discussion of the biological and statistical aspects of the RS-SNP approach. The FDR seems the most relevant summary statistic in this type of analysis as the number of true positives is expected to be a small fraction of the total number of hypotheses tested. More sophisticated scores can be used to measure enrichment instead of the simple indicator function. However, an advantage of the scoring we propose is that it assigns equal weights both to markers strongly associated to CD as well as markers with moderate association, markers with with p-value *P *= 1 × 10^-10 ^and *P *= 1 × 10^-3 ^are treated equally. This property of the score ensures that the enrichment of a gene set is due to the simultaneous presence of many markers with association and not a few with strong association. The methodology also corrects for gene set size automatically.

The linkage disequilibrium (LD) structure is preserved by the proposed method and does not alter the statistical significance of the identified pathways. This is due to the fact that the method uses random permutations of the phenotypic status of the subjects in the sample to assess the significance of the enrichment score. The column permutation procedure does not modify the genotypic profile of the subjects because it limits itself to assign randomly phenotypic states to subjects. The row permutation procedure adopted by the method has the objective of normalizing the enrichment score. This is realized comparing the actual number of markers associated to the phenotype in the gene set with the one obtained by chance. So, the LD structure of a given gene set remains the same under both null and alternative hypothesis. Finally note that the row permutations are only implicitly realized in our approach. This is due to the fact that the number of markers belonging to the gene set and associated to the trait has a hypergeometric distribution. For this reason the computational complexity of RS-SNP is proportional only to the number of column permutations required, that is equal to the inverse of the minimum observable P-value.

From a biological point of view, significant associations were highlighted by RS-SNP analysis between CD and key inflammatory *pathways*. Some of the highlighted pathways were also found to be associated to CD and other inflammatory diseases (rheumatoid arthritis and type I diabetes) by another pathway based method [22]: HSA04630 JAK STAT SIGNALING PATHWAY, HSA04612 ANTIGEN PROCESSING AND PRESENTATION, HSA04514 CELL ADHESION MOLECULES, HSA04650 NATURAL KILLER CELL MEDIATED CYTOTOXICITY and HSA04640 HEMATOPOIETIC CELL LINEAGE. Other pathways such as L6, HSA04940 TYPE I DIABETES MELLITUS, ERK, and IL10 have been associated in the literature to CD [22]. We also found pathway hits for calcium signaling, CREB pathway, IL2 and IL12 which were also found in Torkamani [23] and Wang et al. [9]. These findings are consistent with known functional roles of these pathways in intestinal immune response to microbial infection and injury, signal transduction in response to a variety of extracellular signals including neurotransmitters, hormones, membrane depolarization, and growth, and neurotrophic factors and the exaggerated response observed in CD.

A comparative study of RS-SNP and GENGEN suggests that gene set methods that use both types of null hypotheses may reduce false positives, GENGEN does not randomize with respect to gene set size. It is worth noting that GENGEN found a greater number of significant pathways, but several pathways of these pathways may be false positives. For example, the HSA04810 REGULATION OF ACTIN CYTOSKELETON pathway was found significant by GENGEN. This is a very large pathway composed of 166 genes and 2650 SNPs. Only 32 SNPs are weakly associated (p-value *P *≤ 0.01) to CD and RS-SNP assigned a p-value *P *= 0.82 to this pathway. This type of result is recapitulated with several pathways in the analysis.

## Conclusions

A new method for detecting association of SNP sets to a trait has been proposed. The approach, named RS-SNP, assesses whether the number of SNPs associated to the phenotype and belonging to a given SNP set is statistically significant. Strong signals as well as SNPs weakly associated to the trait are taken into account simultaneously for assessing association of a given SNP set. The proposed method, well founded from a theoretical perspective, is a valuable alternative to other techniques for enrichment analysis of SNP sets. When applied to the CD data set collected by the WTCCC, the method highlighted many relevant pathways which play a key role in CD as well as in other inflammatory diseases.

## Availability and requirements

The RS-SNP approach has been implemented in Matlab in the compute_rs.m file (see additional file 3). Moreover, a combine_rs.m program can be used that allows to combine results obtained by running several times compute_rs.m on the same data, splitting the time-consuming permutation procedure in several blocks. The combine_rs.m program generates a single test statistics for all candidate pathways.

To compute the association of each single SNP with the trait, the compute_association.m program is also enclosed in the RS-SNP package. It allows to perform sample and marker quality controls and then to test the association by choosing the more suitable genetic model.

## Authors' contributions

All the authors conceived the study. AD'A, SM and NA designed the algorithms and conduced the experiments and, together with OP, AL and VA, evaluated and compared the experimental results. All the authors contributed to the drafting of the article.

## Supplementary Material

Additional file 1 **Experimental results of RS-SNP and GENGEN on MSigDB C2 collection**. Tables reporting the experimental results obtained by the proposed method, RS-SNP, and by GENGEN on the MSigDB C2 pathway collection. Click here for file

Additional file 2 **Experimental results of RS-SNP and GENGEN on MSigDB C5 collection**. Tables reporting the experimental results obtained by the proposed method, RS-SNP, and by GENGEN on the MSigDB C5 pathway collection. Click here for file

Additional file 3 **RS-SNP package**. The proposed RS-SNP software is contained in this compressed file, together with: • the help documentation, • example files with the SNP-gene mapping and gene-pathway mapping; • example of input files. Click here for file
