# Supplementary material for: RS-SNP: a random-set method for genome-wide association studies
Source: BMC Genomics. 2011 Mar 30;12:166. doi: 10.1186/1471-2164-12-166 (PMC3079664; doi:10.1186/1471-2164-12-166)
Supplement: Additional file 1 — Experimental results of RS-SNP and GENGEN on MSigDB C2 collection. Tables reporting the experimental results obtained by the proposed method, RS-SNP, and by GENGEN on the MSigDB C2 pathway collection. [file 1471-2164-12-166-S1.PDF]

RS-SNP: a Random-Set method for genome-wide association studies.
Additional file 1 - Experimental results of RS-SNP and GENGEN on MSigDB C2 collection

Annarita D'Addabbo[1] , Orazio Palmieri[2] , Anna Latiano[2] , Vito Annese[2] , Sayan Mukherjee[3] and Nicola Ancona*[1]

[1]Istituto di Studi sui Sistemi Intelligenti per l'Automazione - CNR, Via Amendola 122/D-I, 70126 Bari, Italy,
[2]Ospedale "Casa Sollievo della Sofferenza" IRCCS, Lab Ricerca, Gastroenterologia [3]Departments of Statistical Science, Computer Science, Mathematics, Institute for Genome Science and Policy, Duke University, Durham, NC, USA

Email: Annarita D'Addabbo - daddabbo@ba.issia.cnr.it; Orazio Palmieri - o.palmieri@operapadrepio.it; Anna Latiano - a.latiano@operapadrepio.it; Vito Annese - v.annese@operapadrepio.it; Sayan Mukherjee - sayan@stat.duke.edu; Nicola Ancona*- ancona@ba.issia.cnr.it;

*Corresponding author

Tables

Table 1 - MSigDB C2 CP pathaways rich in weak association signals with CD found by RS-SNP

Table 1: MSigDB C2 CP pathaways rich in weak association signals with CD found by RS-SNP

Pathway	♯ genes	♯ SNPs	p-value	FDR	FWER
HSA04630 JAK STAT SIGNALING PATHWAY	124	883	0.000	0.1513	0.4305
HSA04060 CYTOKINE CYTOKINE RECEPTOR INTERACTION	204	1513	0.0002	0.2460	0.7768
IL3PATHWAY	12	75	0.0003	0.1115	0.1710
IL6PATHWAY	18	123	0.0004	0.1602	0.3990
P35ALZHEIMERSPATHWAY	9	135	0.0005	0.0059	0.0057
HSA01510 NEURODEGENERATIVE DISEASES	35	728	0.0008	0.1348	0.2510
BIOPEPTIDESPATHWAY	33	676	0.0013	0.1251	0.2832
CTLA4PATHWAY	16	93	0.0014	0.2370	0.6257
HSA05010 ALZHEIMERS DISEASE	23	286	0.0018	0.0694	0.0848
HSA04940 TYPE I DIABETES MELLITUS	35	405	0.0019	0.2861	0.9185
ERKPATHWAY	25	310	0.0023	0.2720	0.9189
NKTPATHWAY	25	153	0.0031	0.2966	0.7445
HSA04612 ANTIGEN PROCESSING AND PRESENTATION	55	256	0.0038	0.2915	0.9811
IL18PATHWAY	6	22	0.0051	0.2983	0.9867
TH1TH2PATHWAY	17	126	0.0060	0.2829	0.7640
HSA04514 CELL ADHESION MOLECULES	113	2038	0.0060	0.3128	0.9742
IL4PATHWAY	10	78	0.0062	0.2545	0.8115
NGFPATHWAY	16	105	0.0082	0.2636	0.8894
ERK5PATHWAY	15	96	0.0082	0.2992	0.9649
NO2IL12PATHWAY	14	104	0.0094	0.3099	0.9787
TELPATHWAY	15	348	0.0095	0.2998	0.9880
EPOPATHWAY	14	89	0.0098	0.2721	0.8838
N GLYCAN DEGRADATION	9	65	0.0105	0.3263	0.9649
HSA00511 N GLYCAN DEGRADATION	10	69	0.0112	0.3078	0.9758
HSA00510 N GLYCAN BIOSYNTHESIS	34	390	0.0115	0.2924	0.9832
SA TRKA RECEPTOR	13	105	0.0127	0.3078	0.9468

Table 1 – continued from previous page

Pathway	♯ genes	♯ SNPs	p-value	FDR	FWER
HSA04650 NATURAL KILLER CELL MEDIATED CYTOTOXICITY	103	1120	0.0129	0.3250	0.9988
STRIATED MUSCLE CONTRACTION	32	225	0.0129	0.3584	0.9973
TPOPATHWAY	21	300	0.0142	0.2992	0.9787
FEEDERPATHWAY	8	74	0.0144	0.3237	0.9978
HSA05040 HUNTINGTONS DISEASE	28	242	0.0146	0.2963	0.9484
INSULINPATHWAY	17	124	0.0152	0.2965	0.9801
ST T CELL SIGNAL TRANSDUCTION	40	430	0.0158	0.3314	0.9978
HSA01032 GLYCAN STRUCTURES DEGRADATION	19	287	0.0166	0.2671	0.7759
HSA04660 T CELL RECEPTOR SIGNALING PATHWAY	84	1017	0.0167	0.3228	0.9990
ST STAT3 PATHWAY	10	93	0.0171	0.3176	0.9978
INFLAMPATHWAY	27	107	0.0179	0.3550	0.9999
SMALL LIGAND GPCRS	13	92	0.0195	0.3525	0.9978
SA B CELL RECEPTOR COMPLEXES	20	405	0.0201	0.3196	0.9996
IL12PATHWAY	18	156	0.0220	0.3445	0.9978
DCPATHWAY	19	66	0.0221	0.3744	1.0000
GLEEVECPATHWAY	19	182	0.0223	0.3298	0.9997
HSA00531 GLYCOSAMINOGLYCAN DEGRADATION	12	242	0.0225	0.2405	0.8160
PDGFPATHWAY	22	349	0.0225	0.3268	0.9985
SKP2E2FPATHWAY	9	39	0.0227	0.3551	0.9969
PTENPATHWAY	14	96	0.0228	0.2992	0.9649
HBXPATHWAY	7	67	0.0232	0.2296	0.8233
RNA POLYMERASE	7	34	0.0234	0.3281	0.9997
P27PATHWAY	12	43	0.0235	0.3222	0.9986
IL10PATHWAY	12	99	0.0238	0.3256	0.9989
IGF1RPATHWAY	13	134	0.0243	0.3381	0.9978
IL22BPPATHWAY	12	99	0.0244	0.3401	0.9999
SIG BCR SIGNALING PATHWAY	42	752	0.0252	0.3422	0.9999
ST G ALPHA I PATHWAY	34	775	0.0258	0.3280	0.9997
IGF1PATHWAY	17	172	0.0268	0.3145	0.9996
G1 TO S CELL CYCLE REACTOME	61	281	0.0272	0.3297	0.9997
ST INTERLEUKIN 4 PATHWAY	24	172	0.0277	0.3145	0.9996
IL2PATHWAY	18	190	0.0280	0.3321	0.9997
CREBPATHWAY	24	415	0.0289	0.3309	0.9997
GLYCOSPHINGOLIPID METABOLISM	18	202	0.0295	0.3559	0.9975
HSA04640 HEMATOPOIETIC CELL LINEAGE	68	590	0.0304	0.3595	0.9999
FBW7PATHWAY	8	46	0.0325	0.3308	0.9995
ST DIFFERENTIATION PATHWAY IN PC12 CELLS	36	436	0.0336	0.3319	0.9997
SPRYPATHWAY	15	182	0.0338	0.3526	0.9999
EGFPATHWAY	23	410	0.0346	0.3285	0.9998
GPCRDB CLASS A RHODOPSIN LIKE	143	900	0.0355	0.3603	1.0000
ST PAC1 RECEPTOR PATHWAY	6	70	0.0357	0.3284	0.9997
TCRMOLECULE	2	6	0.0362	0.4068	1.0000
GABAPATHWAY	11	134	0.0368	0.2971	0.9895
CDC25PATHWAY	9	64	0.0368	0.3630	1.0000
ST DICTYOSTELIUM DISCOIDEUM CAMP CHEMOTAXIS	29	542	0.0379	0.3618	1.0000
ST PHOSPHOINOSITIDE 3 KINASE PATHWAY	30	270	0.0383	0.355	0.9999
DREAMPATHWAY	11	73	0.0393	0.3328	0.9997
TCYTOTOXICPATHWAY	9	46	0.0398	0.4360	1.0000
HSA00600 SPHINGOLIPID METABOLISM	33	368	0.0412	0.3269	0.9996
HSA04010 MAPK SIGNALING PATHWAY	222	3274	0.0420	0.3747	1.0000
CYTOKINEPATHWAY	20	80	0.0457	0.3983	1.0000
TCAPOPTOSISPATHWAY	5	35	0.0459	0.3931	1.0000
ST B CELL ANTIGEN RECEPTOR	36	419	0.0464	0.3828	1.0000
IL2RBPATHWAY	28	282	0.0472	0.3840	1.0000
BBCELLPATHWAY	4	3	0.0477	0.3932	1.0000
SA PTEN PATHWAY	16	172	0.0485	0.3356	0.9999
METPATHWAY	30	440	0.0487	0.3555	0.9999
SIG INSULIN RECEPTOR PATHWAY IN CARDIAC MYOCYTES	44	502	0.0488	0.3668	1.0000
RBPATHWAY	13	81	0.0489	0.3921	1.0000
HSA04020 CALCIUM SIGNALING PATHWAY	153	3631	0.0491	0.3870	1.0000

Table 2 - MSigDB C2 CP pathaways rich in weak association signals with CD found by GENGEN

Table 2: MSigDB C2 CP pathaways rich in weak association signals with CD found by GENGEN

Pathway	♯ SNPs	p-value	FDR	FWER
HSA04020 CALCIUM SIGNALING PATHWAY	138	0.0000	0.000	0.0000
HSA04810 REGULATION OF ACTIN CYTOSKELETON	165	0.0000	0.000	0.0000

Table 2 – continued from previous page

Pathway	♯ SNPs	p-value	FDR	FWER
STRIATED MUSCLE CONTRACTION	27	0.0000	0.000	0.0000
HSA04060 CYTOKINE CYTOKINE RECEPTOR INTERACTION	192	0.0000	0.000	0.0003
ST DICTYOSTELIUM DISCOIDEUM CAMP CHEMOTAXIS PATHWAY	28	0.0000	0.000	0.0003
ST GAQ PATHWAY	22	0.0000	0.000	0.0004
ST G ALPHA I PATHWAY	33	0.0000	0.000	0.0005
NO1PATHWAY	26	0.0000	0.000	0.0018
HSA00260 GLYCINE SERINE AND THREONINE METABOLISM	35	0.0000	0.000	0.0022
GLYCINE SERINE AND THREONINE METABOLISM	30	0.0000	0.000	0.003
GPCRDB CLASS A RHODOPSIN LIKE	130	0.0000	0.000	0.0031
HSA04630 JAK STAT SIGNALING PATHWAY	118	0.0000	0.001	0.0111
ST WNT CA2 CYCLIC GMP PATHWAY	19	0.0000	0.001	0.0187
TH1TH2PATHWAY	16	0.0000	0.011	0.2435
GABAPATHWAY	10	0.0000	0.014	0.3023
ST MYOCYTE AD PATHWAY	22	0.0001	0.000	0.0036
SIG CHEMOTAXIS	37	0.0001	0.000	0.0047
SIG BCR SIGNALING PATHWAY	39	0.0001	0.001	0.0075
HSA04510 FOCAL ADHESION	169	0.0001	0.001	0.0113
ST ADRENERGIC	31	0.0001	0.001	0.0197
METHIONINE METABOLISM	11	0.0001	0.067	0.8948
SIG PIP3 SIGNALING IN B LYMPHOCYTES	31	0.0002	0.005	0.0921
HSA00271 METHIONINE METABOLISM	16	0.0002	0.019	0.3902
HSA04940 TYPE I DIABETES MELLITUS	34	0.0003	0.004	0.0795
PEPIPATHWAY	7	0.0004	0.220	1.0000
GPCRDB OTHER	44	0.0005	0.003	0.0461
STARCH AND SUCROSE METABOLISM	28	0.0005	0.010	0.1972
PEPTIDE GPCRS	54	0.0006	0.007	0.1362
HSA04650 NATURAL KILLER CELL MEDIATED CYTOTOXIC-ITY	96	0.0007	0.003	0.0598
HSA04660 T CELL RECEPTOR SIGNALING PATHWAY	81	0.0010	0.008	0.1575
HSA04514 CELL ADHESION MOLECULES	106	0.0010	0.010	0.2026
HSA04640 HEMATOPOIETIC CELL LINEAGE	65	0.0010	0.011	0.2268
IL22BPPATHWAY	12	0.0010	0.082	0.9498
HSA04070 PHOSPHATIDYLINOSITOL SIGNALING SYSTEM	66	0.0012	0.011	0.2194
HSA04912 GNRH SIGNALING PATHWAY	78	0.0012	0.011	0.2205
HSA04540 GAP JUNCTION	76	0.0012	0.012	0.2609
INFLAMPATHWAY	25	0.0015	0.052	0.8040
ST GA12 PATHWAY	20	0.0021	0.023	0.4742
NKTPATHWAY	24	0.0023	0.043	0.7300
IL12PATHWAY	18	0.0025	0.045	0.7584
SMOOTH MUSCLE CONTRACTION	118	0.0029	0.022	0.4428
CALCINEURIN NF AT SIGNALING	78	0.0030	0.023	0.4718
PPARGPATHWAY	7	0.0031	0.190	0.9996
HSA00510 N GLYCAN BIOSYNTHESIS	33	0.0032	0.027	0.5384
HSA04920 ADIPOCYTOKINE SIGNALING PATHWAY	61	0.0033	0.026	0.5447
HSA00970 AMINOACYL TRNA BIOSYNTHESIS	34	0.0046	0.026	0.5518
ASBCELLPATHWAY	7	0.0047	0.295	1.0000
ST T CELL SIGNAL TRANSDUCTION	37	0.0050	0.029	0.5847
IL6PATHWAY	16	0.0054	0.117	0.9899
HSA00520 NUCLEOTIDE SUGARS METABOLISM	4	0.0056	0.392	1.0000
HSA04720 LONG TERM POTENTIATION	56	0.0066	0.052	0.8237
HSA03050 PROTEASOME	17	0.0070	0.255	1.0000
P35ALZHEIMERSPATHWAY	9	0.0075	0.178	0.9989
HSA04662 B CELL RECEPTOR SIGNALING PATHWAY	51	0.0076	0.053	0.8126
HSA00190 OXIDATIVE PHOSPHORYLATION	78	0.0081	0.052	0.8141
TPOPATHWAY	20	0.0094	0.070	0.9086
NO2IL12PATHWAY	14	0.0095	0.176	0.9988
HSA03320 PPAR SIGNALING PATHWAY	59	0.0098	0.065	0.8815
NGFPATHWAY	15	0.0100	0.192	0.9996
SA B CELL RECEPTOR COMPLEXES	18	0.0108	0.078	0.9364
DREAMPATHWAY	11	0.0113	0.304	1.0000
HSA02010 ABC TRANSPORTERS GENERAL	40	0.0120	0.078	0.9402
HSA00500 STARCH AND SUCROSE METABOLISM	73	0.0124	0.075	0.9228
CTLA4PATHWAY	15	0.0124	0.219	1.0000
HSA05040 HUNTINGTONS DISEASE	27	0.0125	0.076	0.9285
HSA04730 LONG TERM DEPRESSION	65	0.0125	0.080	0.945
STEMPATHWAY	14	0.0137	0.355	1.0000
ERKPATHWAY	24	0.0142	0.086	0.9582
CALCIUM REGULATION IN CARDIAC CELLS	113	0.0144	0.098	0.9733
TCRAPATHWAY	7	0.0148	0.261	1.0000
SETPATHWAY	7	0.0160	0.374	1.0000
NUCLEOTIDE SUGARS METABOLISM	3	0.0161	0.430	1.0000
IL17PATHWAY	12	0.0167	0.376	1.0000
ST STAT3 PATHWAY	10	0.0169	0.226	1.0000

Table 2 – continued from previous page

Pathway	♯ SNPs	p-value	FDR	FWER
HSA01510 NEURODEGENERATIVE DISEASES	34	0.0171	0.099	0.9761
PDGFPATHWAY	21	0.0172	0.111	0.984
THELPERPATHWAY	9	0.0172	0.360	1.0000
IL3PATHWAY	10	0.0172	0.359	1.0000
RAC1PATHWAY	20	0.0176	0.113	0.9866
TOLLPATHWAY	27	0.0177	0.110	0.9846
PPARAPATHWAY	41	0.0194	0.105	0.9801
BBCELLPATHWAY	3	0.0213	0.458	1.0000
IL10PATHWAY	12	0.0215	0.262	1.0000
AMINOACYL TRNA BIOSYNTHESIS	20	0.0218	0.116	0.9888
ONE CARBON POOL BY FOLATE	13	0.0248	0.192	0.9996
TCRPATHWAY	35	0.0253	0.148	0.9971
PROTEASOME	12	0.0261	0.414	1.0000
CYTOKINEPATHWAY	20	0.0266	0.357	1.0000
HSA04612 ANTIGEN PROCESSING AND PRESENTATION	52	0.0272	0.131	0.9949
TCAPOPTOSISPATHWAY	5	0.0275	0.453	1.0000
GLUCOCORTICOID MINERALOCORTICOID METABOLISM	6	0.0313	0.427	1.0000
ETSPATHWAY	17	0.0321	0.264	1.0000
FLUMAZENILPATHWAY	7	0.0337	0.197	0.9999
HSA00642 ETHYLBENZENE DEGRADATION	12	0.0345	0.317	1.0000
ST PAC1 RECEPTOR PATHWAY	5	0.0362	0.384	1.0000
RANKLPATHWAY	11	0.0395	0.407	1.0000
FEEDERPATHWAY	8	0.0396	0.328	1.0000
HSA04512 ECM RECEPTOR INTERACTION	77	0.0400	0.206	0.9999
EPOPATHWAY	13	0.0400	0.410	1.0000
IL7PATHWAY	15	0.0404	0.211	1.0000
BIOPEPTIDESPATHWAY	32	0.0408	0.213	1.0000
EGFPATHWAY	22	0.0409	0.219	1.0000
HSA00903 LIMONENE AND PINENE DEGRADATION	25	0.0410	0.223	1.0000
STAT3PATHWAY	7	0.0428	0.414	1.0000
HSA00632 BENZOATE DEGRADATION VIA COA LIGATION	21	0.0435	0.244	1.0000
MSPPATHWAY	4	0.0438	0.459	1.0000
CREBPATHWAY	24	0.0447	0.218	1.0000
HSA00670 ONE CARBON POOL BY FOLATE	15	0.0447	0.251	1.0000
BCRPATHWAY	28	0.0458	0.222	1.0000
PELP1PATHWAY	5	0.0468	0.389	1.0000
ST ERK1 ERK2 MAPK PATHWAY	23	0.0475	0.221	1.0000
RHOPATHWAY	21	0.0482	0.220	1.0000
ST DIFFERENTIATION PATHWAY IN PC12 CELLS	33	0.0496	0.220	1.0000
HEME BIOSYNTHESIS	7	0.0497	0.459	1.0000
PGC1APATHWAY	21	0.0500	0.222	1.0000

Table 3 - Pathways in MSigDB C2 CP collection identified by the two methods with p-value $P \leq 0.05$ and the corresponding FDR, in alphabetical order

Table 3: Pathways in MSigDB C2 CP collection identified by the two methods with p-value ≤ 0.05 and the corresponding FDR, in alphabetical order

			RS-SNP		GSEA-SNP	
Pathway	♯ genes	♯ SNPs	p-value	FDR	p-value	FDR
BBCELLPATHWAY	4	35	0.0477	0.3931	0.0213	0.458
BIOPEPTIDESPATHWAY	33	676	0.0013	0.1251	0.0408	0.213
CREBPATHWAY	24	415	0.0289	0.3309	0.0447	0.218
CTLA4PATHWAY	16	93	0.0014	0.2370	0.0124	0.219
CYTOKINEPATHWAY	20	80	0.0457	0.3983	0.0266	0.357
DREAMPATHWAY	11	73	0.0393	0.3328	0.0113	0.304
EGFPATHWAY	23	410	0.0346	0.3285	0.0409	0.219
EPOPATHWAY	14	89	0.0098	0.2721	0.0400	0.410
ERKPATHWAY	25	310	0.0023	0.2720	0.0142	0.086
FEEDERPATHWAY	8	74	0.0144	0.3237	0.0396	0.328
GABAPATHWAY	11	134	0.0368	0.2971	0.0000	0.014
GPCRDB CLASS A RHODOPSIN LIKE	143	900	0.0355	0.3603	0.0000	0.000
HSA00510 N GLYCAN BIOSYNTHESIS	34	390	0.0115	0.2924	0.0032	0.027
HSA01510 NEURODEGENERATIVE DISEASES	35	728	0.0008	0.1348	0.0171	0.099
HSA04020 CALCIUM SIGNALING PATHWAY	153	3631	0.0491	0.3870	0.0000	0.000
HSA04060 CYTOKINE CYTOKINE RECEPTOR INTERACTION	204	1513	0.0002	0.2457	0.0000	0.000
HSA04514 CELL ADHESION MOLECULES	113	2038	0.0060	0.3128	0.0010	0.010
HSA04612 ANTIGEN PROCESSING AND PRESENTATION	55	256	0.0038	0.2915	0.0272	0.131

Table 3 – continued from previous page

			RS-SNP		GSEA-SNP	
HSA04630 JAK STAT SIGNALING PATHWAY	124	883	0.0000	0.1513	0.0000	0.001
HSA04640 HEMATOPOIETIC CELL LINEAGE	68	590	0.0304	0.3595	0.0010	0.011
HSA04650 NATURAL KILLER CELL MEDIATED CYTOTOXICITY	103	1120	0.0129	0.3250	0.0007	0.003
HSA04660 T CELL RECEPTOR SIGNALING PATHWAY	84	1017	0.0167	0.3228	0.0010	0.008
HSA04940 TYPE I DIABETES MELLITUS	35	405	0.0019	0.2861	0.0003	0.004
HSA05040 HUNTINGTONS DISEASE	28	242	0.0146	0.2963	0.0125	0.076
IL10PATHWAY	12	99	0.0238	0.3256	0.0215	0.262
IL12PATHWAY	18	156	0.022	0.3445	0.0025	0.045
IL22BPPATHWAY	12	99	0.0244	0.3401	0.001	0.082
IL3PATHWAY	12	75	0.0003	0.1115	0.0172	0.359
IL6PATHWAY	18	123	0.0004	0.1602	0.0054	0.117
INFLAMPATHWAY	27	107	0.0179	0.3550	0.0015	0.052
NGFPATHWAY	16	105	0.0082	0.2636	0.0100	0.192
NKTPATHWAY	25	153	0.0031	0.2966	0.0023	0.043
NO2IL12PATHWAY	14	104	0.0094	0.3099	0.0095	0.176
P35ALZHEIMERSPATHWAY	9	135	0.0005	0.0059	0.0075	0.178
PDGFPATHWAY	22	349	0.0225	0.3268	0.0172	0.111
SA B CELL RECEPTOR COMPLEXES	20	405	0.0201	0.3197	0.0108	0.078
SIG BCR SIGNALING PATHWAY	42	752	0.0252	0.3422	0.0001	0.001
ST DICTYOSTELIUM DISCOIDEUM CAMP CHEMOTAXIS PATHWAY	29	542	0.0379	0.3618	0.0000	0.000
ST DIFFERENTIATION PATHWAY IN PC12 CELLS	36	43 6	0.0336	0.3319	0.0496	0.220
ST G ALPHA I PATHWAY	34	775	0.0258	0.3280	0.0000	0.000
ST PAC1 RECEPTOR PATHWAY	6	70	0.0357	0.3284	0.0362	0.384
ST STAT3 PATHWAY	10	93	0.0171	0.3176	0.0169	0.226
ST T CELL SIGNAL TRANSDUCTION	40	430	0.0158	0.3314	0.005	0.029
STRIATED MUSCLE CONTRACTION	32	225	0.0129	0.3584	0.0000	0.000
TCAPOPTOSISPATHWAY	5	35	0.0459	0.3931	0.0275	0.453
TH1TH2PATHWAY	17	126	0.0060	0.2829	0.0000	0.011
TPOPATHWAY	21	300	0.0142	0.2992	0.0094	0.070

Discussion

GENGEN assigns only one SNP (the one with the best p-value) to each gene and so it considers only 166 SNPs, including 14 markers weakly associated and 4 markers (respectively on IQGAP2, ITGA3,MLCK and VAV2 genes) having p-value $P \leq 10^{-7}$. If we compute RS-SNP considering the GENGEN mapping 1 gene - 1 SNP, we have a total of $m = 3892$ genes and $y = 441$ significant genes and this pathway is no significant (p-value $P = 0.84$). ST GAQ PATHWAY shows p-value = 0 in the GENGEN analysis, instead it has p-value $P = 0.40$ in RS-SNP study. This pathway is composed of 22 genes and 379 SNPs on them. Only 6 markers on 3 different genes (DAG1, ITPR1 and ITPR2) are weakly associated with CD (p-value $P = 0.52$ in RS-SNP with GENGEN mapping). The GENGEN p-value is probably due to the intronic SNP on ITPR2 having p-value highly significant. Analogously, NO1PATHWAY showed p-value $P = 0$ in the GENGEN analysis, instead it has p-value $P = 0.61$ in RS-SNP study. This pathway is composed of 26 genes and 734 SNPs. Only 9 markers on 3 different genes (PRKG1, RYR2 and TNNI1) are weakly associated with CD. In particular, PRKG1 and RYR2 are large genes harbouring respectively 269 and 164 markers mapped on Affymetrix GeneChip 500K, but having only 5 and 3 SNPs weakly associated with CD ($9 * 10^{-3} \leq$ p-value ≤ 0.01). Only one marker on TNNI1 is highly associated. In particular, performing RS-SNP with GENGEN gene-SNP mapping this pathway has p-value $P = 0.84$. HSA00260 GLYCINE

SERINE AND THREONINE METABOLISM pathway and GLYCINE SERINE AND THREONINE METABOLISM pathway are very similar. The associated markers are on AMT, BHMT, CHDH and PLCG2 genes, where the last one is only in the GLYCINE SERINE AND THREONINE METABOLISM pathway. On the whole, the first pathway has 6 markers weakly associated (RS-SNP GENGEN like mapping p-value $P = 0.41$), the latter 9 SNPs, but they are composed of more then 200 markers (RS -SNP GENGEN like mapping p-value $P = 0.21$). Among the 67 pathways significant for GENGEN but not significant in the RS-SNP analysis in the analysis of C2 CP collection, only 6 pathways, i.e. ASBCELLPATHWAY, SETPATHWAY, AMINOACYL TRNA BIOSYNTHESIS, PPARGPATHWAY, IL17PATHWAY and PELP1PATHWAY, are significant if we compute the association by RS-SNP and by using the GENGEN 1 gene-1 SNP mapping. In these pathways, each significant gene harbours 1 (or no more than 2) significant makers. This occurrence makes this pathway not significant if we consider the traditional mapping used in RS-SNP, but it makes them significant if the GENGEN mapping is considered. Similar considerations hold for the experimental results of the two methods on the MSigDB C5 collection.
