# Supplementary material for: RS-SNP: a random-set method for genome-wide association studies
Source: BMC Genomics. 2011 Mar 30;12:166. doi: 10.1186/1471-2164-12-166 (PMC3079664; doi:10.1186/1471-2164-12-166)
Supplement: Additional file 2 — Experimental results of RS-SNP and GENGEN on MSigDB C5 collection. Tables reporting the experimental results obtained by the proposed method, RS-SNP, and by GENGEN on the MSigDB C5 pathway collection. [file 1471-2164-12-166-S2.PDF]

RS-SNP: a Random-Set method for genome-wide association studies.
Additional file 2 - Experimental results of RS-SNP and GENGEN on MSigDB C5 collection

Annarita D'Addabbo[1] , Orazio Palmieri[2] , Anna Latiano[2] , Vito Annese[2] , Sayan Mukherjee[3] and Nicola Ancona*[1]

[1]Istituto di Studi sui Sistemi Intelligenti per l'Automazione - CNR, Via Amendola 122/D-I, 70126 Bari, Italy,
[2]Ospedale "Casa Sollievo della Sofferenza" IRCCS, Lab Ricerca, Gastroenterologia [3]Departments of Statistical Science, Computer Science, Mathematics, Institute for Genome Science and Policy, Duke University, Durham, NC, USA

Email: Annarita D'Addabbo - daddabbo@ba.issia.cnr.it; Orazio Palmieri - o.palmieri@operapadrepio.it; Anna Latiano - a.latiano@operapadrepio.it; Vito Annese - v.annese@operapadrepio.it; Sayan Mukherjee - sayan@stat.duke.edu; Nicola Ancona*- ancona@ba.issia.cnr.it;

*Corresponding author

Tables

Table 1 - MSigDB C5 pathaways rich in weak association signals with CD found by RS-SNP

Table 1: MSigDB C5 pathaways rich in weak association signals with CD found by RS-SNP

Pathway	♯ genes	♯ SNPs	p-value	FDR	FWER
MEMBRANE	1683	25238	0.0002	0.2098	0.9844
INTERLEUKIN RECEPTOR ACTIVITY	18	143	0.0011	0.1543	0.8416
MICROTUBULE BASED PROCESS	75	866	0.0011	0.0880	0.5977
POSITIVE REGULATION OF CYTOKINE PRODUCTION	14	103	0.0012	0.1594	0.9208
INTERLEUKIN BINDING	21	158	0.0013	0.1666	0.9014
PLASMA MEMBRANE	1213	20009	0.0013	0.2464	0.9959
CYTOKINE BINDING	38	262	0.0014	0.1797	0.9599
POSITIVE REGULATION OF PROTEIN METABOLIC PROCESS	65	474	0.0014	0.0647	0.2848
REGULATION OF CELLULAR COMPONENT ORGANIZATION AND BIOGENESIS	108	1529	0.0014	0.1676	0.9155
REGULATION OF CYTOSKELETON ORGANIZATION AND BIOGENESIS	26	345	0.0014	0.0626	0.1706
ENZYME REGULATOR ACTIVITY	282	3866	0.0015	0.2099	0.9834
REGULATION OF ORGANELLE ORGANIZATION AND BIOGENESIS	35	397	0.0019	0.0647	0.2444
TRANSFERASE ACTIVITY TRANSFERRING PENTOSYL GROUPS	17	176	0.0019	0.1427	0.7849
GENERATION OF NEURONS	74	2023	0.0020	0.1687	0.9122
RECEPTOR BINDING	315	3004	0.0020	0.2381	0.9974
GROWTH CONE	10	338	0.0022	0.0565	0.1786
SITE OF POLARIZED GROWTH	11	345	0.0022	0.0528	0.1886
INTRINSIC TO PLASMA MEMBRANE	841	12909	0.0024	0.2465	0.9982
HEMATOPOIETIN INTERFERON CLASS D200 DOMAIN CYTOKINE RECEPTOR ACTIVITY	30	317	0.0025	0.1912	0.9700
NEUROGENESIS	82	2099	0.0025	0.1665	0.9366
NEURON DIFFERENTIATION	68	1952	0.0025	0.1572	0.9235

Table 1 – continued from previous page

Pathway	♯ genes	♯ SNPs	p-value	FDR	FWER
AXONOGENESIS	36	1218	0.0026	0.1487	0.8084
ENZYME INHIBITOR ACTIVITY	96	698	0.0026	0.2353	0.9920
MULTI ORGANISM PROCESS	142	868	0.0026	0.2381	0.9970
INTRACELLULAR NON MEMBRANE BOUND ORGANELLE	551	5840	0.0027	0.2389	0.9960
NEURITE DEVELOPMENT	46	1268	0.0027	0.1521	0.8273
NON MEMBRANE BOUND ORGANELLE	551	5840	0.0027	0.2389	0.9960
REGULATION OF PROTEIN METABOLIC PROCESS	145	1017	0.0028	0.1541	0.8598
CELLULAR MORPHOGENESIS DURING DIFFERENTIATION	42	1280	0.0029	0.1591	0.8591
ENZYME BINDING	158	2083	0.0029	0.1780	0.9612
INTEGRAL TO PLASMA MEMBRANE	833	12627	0.0033	0.2499	0.9993
POSITIVE REGULATION OF CELLULAR COMPONENT ORGANIZATION AND BIOGENESIS	31	426	0.0035	0.0568	0.3425
NEURON DEVELOPMENT	53	1411	0.0036	0.1630	0.9205
INTRACELLULAR ORGANELLE PART	1029	8715	0.0037	0.2549	0.9998
ORGANELLE PART	1032	8724	0.0037	0.2541	0.9998
POSITIVE REGULATION OF CELLULAR PROTEIN METABOLIC PROCESS	63	450	0.0038	0.0634	0.3893
HYDROLASE ACTIVITY HYDROLYZING O GLYCOSYL COMPOUNDS	25	369	0.0040	0.0619	0.3991
NUCLEUS	1215	10615	0.0042	0.2484	0.9997
CYTOKINE ACTIVITY	90	478	0.0044	0.2031	0.9784
HYDROLASE ACTIVITY ACTING ON GLYCOSYL BONDS	34	424	0.0044	0.0803	0.5311
NUCLEAR UBIQUITIN LIGASE COMPLEX	8	79	0.0049	0.1370	0.7595
REGULATION OF CELLULAR PROTEIN METABOLIC PROCESS	136	947	0.0051	0.1649	0.9183
REGULATION OF CYTOKINE PRODUCTION	23	185	0.0051	0.2523	0.9998
REGULATION OF PROTEIN SECRETION	18	179	0.0051	0.1766	0.9530
INTRINSIC TO MEMBRANE	1137	16350	0.0058	0.2610	0.9999
MICROTUBULE ASSOCIATED COMPLEX	44	417	0.0063	0.0747	0.4741
MICROTUBULE CYTOSKELETON ORGANIZATION AND BIOGENESIS	32	209	0.0071	0.0531	0.1231
RAS GUANYL NUCLEOTIDE EXCHANGE FACTOR ACTIVITY	17	550	0.0073	0.2467	0.9981
REGULATION OF ANATOMICAL STRUCTURE MORPHOGENESIS	24	417	0.0074	0.0801	0.5133
SERINE TYPE ENDOPEPTIDASE INHIBITOR ACTIVITY	24	209	0.0074	0.2069	0.9850
IMMUNE RESPONSE	190	1404	0.0075	0.2520	0.9999
MICROTUBULE CYTOSKELETON	143	1179	0.0078	0.1793	0.9573
REGULATION OF AXONOGENESIS	9	269	0.0081	0.0660	0.2700
MICROTUBULE POLYMERIZATION OR DEPOLYMERIZATION	10	101	0.0083	0.0523	0.0268
REGULATION OF NEUROGENESIS	13	296	0.0083	0.0581	0.3318
STRUCTURAL MOLECULE ACTIVITY	195	1922	0.0085	0.2369	0.9961
CHROMOSOMAL PART	80	439	0.0087	0.2464	0.9982
SMALL GTPASE REGULATOR ACTIVITY	58	988	0.0087	0.2538	0.9998
PLASMA MEMBRANE PART	987	16216	0.0088	0.2689	0.9999
REGULATION OF PROTEIN POLYMERIZATION	10	127	0.0089	0.0694	0.0648
GUANYL NUCLEOTIDE EXCHANGE FACTOR ACTIVITY	42	1177	0.0090	0.2476	0.9990
MICROTUBULE	32	245	0.0090	0.0562	0.3084
MICROTUBULE BINDING	32	346	0.0090	0.0841	0.5646
AXON	10	113	0.0091	0.0471	0.0657
CYTOSOL	181	1826	0.0091	0.2424	0.9968
POSITIVE REGULATION OF CYTOSKELETON ORGANIZATION AND BIOGENESIS	10	136	0.0091	0.0414	0.0755
SH3 DOMAIN BINDING	13	242	0.0091	0.0585	0.2997
INTEGRAL TO MEMBRANE	1127	16058	0.0092	0.2687	0.9999
PROTEIN POLYMERIZATION	17	241	0.0093	0.0627	0.2971
PROTEASE INHIBITOR ACTIVITY	39	286	0.0101	0.2499	0.9990
REGULATION OF INTERFERON GAMMA BIOSYNTHETIC PROCESS	8	29	0.0102	0.2711	0.9999
PROTEIN METABOLIC PROCESS	1052	12645	0.0103	0.2668	0.9999
GTPASE REGULATOR ACTIVITY	111	2514	0.0105	0.2486	0.9999
DETECTION OF EXTERNAL STIMULUS	22	145	0.0107	0.2393	0.9934
NEURON PROJECTION	19	571	0.0108	0.1659	0.8942
STRUCTURAL CONSTITUENT OF MUSCLE	30	437	0.0109	0.2484	0.9994
STRUCTURAL CONSTITUENT OF CYTOSKELETON	48	619	0.0111	0.1649	0.9392
CYTOSKELETON ORGANIZATION AND BIOGENESIS	188	2673	0.0112	0.2498	0.9993
RESPONSE TO BIOTIC STIMULUS	105	560	0.0114	0.2496	0.9995
PROTEIN SECRETION	27	316	0.0118	0.2503	0.9999
RESPONSE TO OTHER ORGANISM	72	367	0.0118	0.2645	0.9999
IMMUNE SYSTEM PROCESS	277	2492	0.0120	0.2631	0.9999
REGULATION OF TRANSCRIPTION FACTOR ACTIVITY	35	268	0.0125	0.2492	0.9992
TUBULIN BINDING	45	517	0.0126	0.1661	0.8842
LIPOPROTEIN BINDING	16	656	0.0130	0.2135	0.9833
MAINTENANCE OF CELLULAR PROTEIN LOCALIZATION	8	42	0.0134	0.2453	0.9979

Table 1 – continued from previous page

Pathway	♯ genes	♯ SNPs	p-value	FDR	FWER
PROTEIN DOMAIN SPECIFIC BINDING	61	1402	0.0134	0.2408	0.9976
DETECTION OF BIOTIC STIMULUS	10	67	0.0135	0.2402	0.9970
MAINTENANCE OF CELLULAR LOCALIZATION	9	44	0.0137	0.2454	0.9986
LYSOSOME	50	399	0.0140	0.2503	0.9998
LYTIC VACUOLE	50	399	0.0140	0.2503	0.9998
INTERFERON GAMMA BIOSYNTHETIC PROCESS	9	34	0.0142	0.2912	1.0000
PEPTIDYL AMINO ACID MODIFICATION	57	541	0.0145	0.2682	0.9999
ANATOMICAL STRUCTURE MORPHOGENESIS	326	4717	0.0147	0.2762	1.0000
CHEMOKINE ACTIVITY	32	124	0.0147	0.2507	0.9999
CYTOSKELETON	333	4130	0.0147	0.2630	0.9999
MAINTENANCE OF PROTEIN LOCALIZATION	9	48	0.0147	0.2473	0.9995
INTERLEUKIN 1 SECRETION	8	95	0.0148	0.2490	0.9993
CYTOSKELETAL PART	215	2270	0.0149	0.2511	0.9998
MEMBRANE PART	1413	20710	0.0154	0.3162	1.0000
SECRETIN LIKE RECEPTOR ACTIVITY	10	73	0.0156	0.2483	0.9986
CHEMOKINE RECEPTOR BINDING	33	130	0.0157	0.2510	0.9999
MICROBODY MEMBRANE	9	59	0.0159	0.2448	0.9990
PEROXISOMAL MEMBRANE	9	59	0.0159	0.2448	0.9990
DEFENSE RESPONSE	230	1620	0.0166	0.2959	1.0000
INTERFERON GAMMA PRODUCTION	11	37	0.0170	0.3169	1.0000
GUANYL NUCLEOTIDE BINDING	42	266	0.0177	0.2425	0.9999
REGULATION OF DNA BINDING	42	296	0.0180	0.2450	0.9999
MICROBODY PART	10	68	0.0181	0.2449	0.9999
PEROXISOMAL PART	10	68	0.0181	0.2449	0.9999
NEGATIVE REGULATION OF SECRETION	10	87	0.0190	0.2482	0.9981
MONOCARBOXYLIC ACID TRANSPORT	8	67	0.0196	0.2479	0.9999
PROTEIN OLIGOMERIZATION	35	513	0.0199	0.2660	0.9999
RESPONSE TO BACTERIUM	26	163	0.0199	0.2711	0.9999
CELL DEVELOPMENT	491	7007	0.0206	0.2993	1.0000
POSITIVE REGULATION OF DEVELOPMENTAL PROCESS	189	2382	0.0218	0.2691	0.9999
DEFENSE RESPONSE TO BACTERIUM	21	144	0.0224	0.2698	0.9999
TRANSMEMBRANE RECEPTOR ACTIVITY	353	6830	0.0224	0.3238	1.0000
PROTEIN COMPLEX	705	7498	0.0225	0.3213	1.0000
I KAPPAB KINASE NF KAPPAB CASCADE	101	666	0.0228	0.3158	1.0000
REGULATION OF METABOLIC PROCESS	694	6998	0.0228	0.3236	1.0000
PEROXISOME ORGANIZATION AND BIOGENESIS	13	81	0.0231	0.2679	0.9999
MAINTENANCE OF LOCALIZATION	17	76	0.0235	0.3142	1.0000
REGULATION OF SECRETION	32	346	0.0243	0.2670	0.9999
AMINOPEPTIDASE ACTIVITY	14	240	0.0246	0.2044	0.9772
SIGNAL SEQUENCE BINDING	13	106	0.0246	0.2434	0.9999
CHROMOSOME	102	586	0.0250	0.2904	1.0000
LYSOSOMAL MEMBRANE	9	61	0.0250	0.2361	0.9915
POSITIVE REGULATION OF MULTICELLULAR ORGANISMAL PROCESS	58	444	0.0250	0.3126	1.0000
REGULATION OF ACTIN FILAMENT LENGTH	11	173	0.0250	0.2500	0.9992
VACUOLAR MEMBRANE	10	63	0.0250	0.2389	0.9940
VACUOLAR PART	11	64	0.0251	0.2423	0.9949
VACUOLE	55	444	0.0253	0.2699	0.9999
AXON GUIDANCE	19	770	0.0256	0.2657	0.9999
COFACTOR TRANSPORTER ACTIVITY	9	60	0.0256	0.2668	0.9999
POSITIVE REGULATION OF CYTOKINE SECRETION	7	89	0.0260	0.2503	0.9999
REGULATION OF CELLULAR COMPONENT SIZE	12	181	0.0260	0.2508	0.9997
REGULATION OF BLOOD PRESSURE	18	99	0.0261	0.2674	0.9999
REGULATION OF DEVELOPMENTAL PROCESS	382	5022	0.0263	0.3173	1.0000
CELLULAR PROTEIN METABOLIC PROCESS	954	11291	0.0266	0.3297	1.0000
POSITIVE REGULATION OF METABOLIC PROCESS	214	2529	0.0268	0.3185	1.0000
POSITIVE REGULATION OF CYTOKINE BIOSYNTHETIC PROCESS	19	80	0.0269	0.3268	1.0000
HEMATOPOIETIN INTERFERON CLASS D200 DOMAIN CYTOKINE RECEPTOR BINDING	22	72	0.0270	0.3060	1.0000
POSITIVE REGULATION OF PROTEIN SECRETION	8	94	0.0270	0.2658	0.9999
GTPASE ACTIVITY	82	666	0.0280	0.2961	1.0000
TRANSFERASE ACTIVITY TRANSFERRING GLYCOSYL GROUPS	88	1231	0.0280	0.3218	1.0000
UBIQUITIN LIGASE COMPLEX	22	193	0.0281	0.2455	0.9999
PROTEIN AMINO ACID O LINKED GLYCOSYLATION	13	171	0.0286	0.2694	0.9999
COFACTOR TRANSPORT	10	68	0.0287	0.2909	1.0000
ACTIVATION OF NF KAPPAB TRANSCRIPTION FACTOR	16	188	0.0290	0.2983	1.0000
POSITIVE REGULATION OF TRANSLATION	27	173	0.0292	0.3577	1.0000
CELL PROJECTION	102	2041	0.0293	0.2697	0.9999
KINESIN COMPLEX	12	74	0.0294	0.3144	1.0000
RECEPTOR ACTIVITY	498	8905	0.0296	0.3497	1.0000
ADENYLATE CYCLASE ACTIVATION	15	114	0.0299	0.2664	0.9999
REGULATION OF CYTOKINE SECRETION	13	102	0.0302	0.2676	0.9999

Table 1 – continued from previous page

Pathway	♯ genes	♯ SNPs	p-value	FDR	FWER
CELLULAR MACROMOLECULE METABOLIC PROCESS	967	11400	0.0304	0.3415	1.0000
REGULATION OF CATABOLIC PROCESS	15	79	0.0319	0.3256	1.0000
REGULATION OF CELLULAR METABOLIC PROCESS	684	6937	0.0329	0.3453	1.0000
NEGATIVE REGULATION OF INTRACELLULAR TRANSPORT	10	71	0.0331	0.3006	1.0000
MACROMOLECULAR COMPLEX	814	7968	0.0336	0.3519	1.0000
PEPTIDASE ACTIVITY	157	1482	0.0336	0.3280	1.0000
G PROTEIN COUPLED RECEPTOR BINDING	41	179	0.0337	0.3253	1.0000
NERVOUS SYSTEM DEVELOPMENT	308	7100	0.0349	0.3383	1.0000
POSITIVE REGULATION OF TRANSCRIPTION FACTOR ACTIVITY	22	209	0.0349	0.3253	1.0000
NEGATIVE REGULATION OF TRANSCRIPTION FACTOR ACTIVITY	12	59	0.0350	0.2727	0.9999
ORGANELLE ORGANIZATION AND BIOGENESIS	417	4299	0.0353	0.3310	1.0000
RESPIRATORY GASEOUS EXCHANGE	13	97	0.0354	0.3196	1.0000
REGULATION OF BINDING	51	400	0.0359	0.3238	1.0000
POSITIVE REGULATION OF DNA BINDING	24	214	0.0365	0.3261	1.0000
POSITIVE REGULATION OF SECRETION	16	128	0.0366	0.3225	1.0000
RESPONSE TO EXTERNAL STIMULUS	269	2798	0.0368	0.3682	1.0000
CELL MIGRATION	85	1918	0.0374	0.3400	1.0000
GTP BINDING	41	238	0.0386	0.3279	1.0000
CYSTEINE TYPE PEPTIDASE ACTIVITY	51	310	0.0393	0.3779	1.0000
CYTOKINE SECRETION	15	162	0.0405	0.3375	1.0000
POSITIVE REGULATION OF BINDING	26	228	0.0409	0.3510	1.0000
MRNA BINDING	16	97	0.0410	0.3714	1.0000
RHO GUANYL NUCLEOTIDE EXCHANGE FACTOR ACTIVITY	12	487	0.0411	0.3384	1.0000
EXOPEPTIDASE ACTIVITY	32	399	0.0416	0.2624	0.9999
ADAPTIVE IMMUNE RESPONSE GO 0002460	21	132	0.0423	0.3918	1.0000
ADAPTIVE IMMUNE RESPONSE	22	135	0.0434	0.3992	1.0000
HYDROLASE ACTIVITY ACTING ON CARBON NITROGEN BUT NOT PEPTIDE BONDS IN LINEAR AMIDES	18	59	0.0435	0.4050	1.0000
POSITIVE REGULATION OF CELLULAR METABOLIC PROCESS	208	2503	0.0438	0.3697	1.0000
POSITIVE REGULATION OF CELLULAR PROCESS	585	6716	0.0438	0.3763	1.0000
REGULATION OF BIOLOGICAL QUALITY	361	4619	0.0445	0.3710	1.0000
ACETYLGALACTOSAMINYLTRANSFERASE ACTIVITY	11	201	0.0453	0.3146	1.0000
NEGATIVE REGULATION OF DNA BINDING	14	81	0.0453	0.3241	1.0000
SMALL CONJUGATING PROTEIN SPECIFIC PROTEASE ACTIVITY	9	42	0.0456	0.4404	1.0000
NEGATIVE REGULATION OF BINDING	15	85	0.0463	0.3397	1.0000
PROTEIN HOMOOLIGOMERIZATION	19	330	0.0471	0.3533	1.0000
REGULATION OF PROTEIN IMPORT INTO NUCLEUS	13	107	0.0482	0.3961	1.0000
MONOCARBOXYLIC ACID TRANSMEMBRANE TRANSPORTER ACTIVITY	11	123	0.0488	0.3707	1.0000
G PROTEIN SIGNALING ADENYLATE CYCLASE ACTIVATING PATHWAY	19	146	0.0489	0.3166	1.0000
PROTEIN KINASE BINDING	52	494	0.0497	0.3713	1.0000
POSITIVE REGULATION OF I KAPPAB KINASE NF KAPPAB CASCADE	77	465	0.0500	0.4094	1.0000

Table 2 - MSigDB C5 pathways rich in weak association signals with CD found by GENGEN

Table 2: MSigDB C5 pathways rich in weak association signals with CD found by GENGEN

Pathway	♯ SNPs	p-value	FDR	FWER
METAL ION TRANSMEMBRANE TRANSPORTER ACTIVITY	125	0.0000	0.000	0.0001
STEROID METABOLIC PROCESS	55	0.0000	0.000	0.0001
RECEPTOR ACTIVITY	463	0.0000	0.000	0.0001
ENDOPLASMIC RETICULUM	226	0.0000	0.000	0.0002
CELLULAR LIPID METABOLIC PROCESS	210	0.0000	0.000	0.0002
LIPID METABOLIC PROCESS	267	0.0000	0.000	0.0004
CATION TRANSMEMBRANE TRANSPORTER ACTIVITY	179	0.0000	0.000	0.0016
ION TRANSMEMBRANE TRANSPORTER ACTIVITY	227	0.0000	0.000	0.0019
LIGAND GATED CHANNEL ACTIVITY	33	0.0000	0.000	0.0033
SIGNAL TRANSDUCTION	1299	0.0000	0.001	0.0052
GATED CHANNEL ACTIVITY	102	0.0000	0.001	0.0130
EXCITATORY EXTRACELLULAR LIGAND GATED ION CHANNEL ACTIVITY	18	0.0000	0.001	0.0135
EXTRACELLULAR LIGAND GATED ION CHANNEL ACTIVITY	18	0.0000	0.001	0.0135

Table 2 – continued from previous page

Pathway	♯ SNPs	p-value	FDR	FWER
ALCOHOL METABOLIC PROCESS	69	0.0000	0.001	0.0139
TRANSFERASE ACTIVITY TRANSFERRING ONE CARBON GROUPS	32	0.0000	0.001	0.0157
ION CHANNEL ACTIVITY	125	0.0000	0.001	0.0167
AMINE RECEPTOR ACTIVITY	25	0.0000	0.001	0.0179
METHYLTRANSFERASE ACTIVITY	31	0.0000	0.001	0.0191
TRANSMEMBRANE RECEPTOR ACTIVITY	330	0.0000	0.001	0.0207
PLASMA MEMBRANE	1128	0.0000	0.001	0.0228
CATION CHANNEL ACTIVITY	102	0.0000	0.002	0.0259
PEPTIDASE ACTIVITY	149	0.0000	0.002	0.0412
INORGANIC CATION TRANSMEMBRANE TRANSPORTER ACTIVITY	47	0.0000	0.006	0.1074
S ADENOSYLMETHIONINE DEPENDENT METHYLTRANSFERASE ACTIVITY	21	0.0000	0.006	0.1172
RHODOPSIN LIKE RECEPTOR ACTIVITY	94	0.0000	0.007	0.1335
DETECTION OF EXTERNAL STIMULUS	19	0.0000	0.007	0.1382
SEROTONIN RECEPTOR ACTIVITY	10	0.0000	0.008	0.1662
DETECTION OF BIOTIC STIMULUS	9	0.0000	0.125	1.0000
DIGESTION	37	0.0001	0.001	0.0045
SUBSTRATE SPECIFIC CHANNEL ACTIVITY	131	0.0001	0.003	0.0526
PROTEIN METABOLIC PROCESS	979	0.0001	0.006	0.1052
G PROTEIN COUPLED RECEPTOR ACTIVITY	140	0.0001	0.007	0.1527
RESPONSE TO BACTERIUM	22	0.0001	0.014	0.3505
REGULATION OF CATABOLIC PROCESS	14	0.0001	0.074	0.9666
MEMBRANE	1563	0.0002	0.003	0.0654
DI TRI VALENT INORGANIC CATION TRANSMEMBRANE TRANSPORTER ACTIVITY	18	0.0002	0.007	0.1518
TRANSMEMBRANE TRANSPORTER ACTIVITY	308	0.0002	0.007	0.1664
RECEPTOR BINDING	290	0.0003	0.002	0.0355
SUBSTRATE SPECIFIC TRANSPORTER ACTIVITY	318	0.0004	0.013	0.2887
DEFENSE RESPONSE TO BACTERIUM	17	0.0004	0.028	0.6249
REGULATION OF CELL PROLIFERATION	250	0.0005	0.010	0.2159
CYTOPLASM	1665	0.0005	0.017	0.4089
ENZYME BINDING	148	0.0006	0.011	0.2477
CARBOHYDRATE BINDING	58	0.0007	0.012	0.2788
ANGIOGENESIS	38	0.0007	0.014	0.3429
LIPID TRANSPORT	21	0.0007	0.028	0.6476
VITAMIN TRANSPORT	12	0.0007	0.031	0.6919
COFACTOR TRANSPORTER ACTIVITY	8	0.0007	0.160	1.0000
CELLULAR LIPID CATABOLIC PROCESS	29	0.0009	0.014	0.3266
SUBSTRATE SPECIFIC TRANSMEMBRANE TRANSPORTER ACTIVITY	281	0.0009	0.014	0.3416
POSITIVE REGULATION OF CYTOKINE PRODUCTION	13	0.0009	0.072	0.9606
POSITIVE REGULATION OF CELLULAR PROCESS	548	0.0012	0.014	0.3238
REGULATION OF LIPID METABOLIC PROCESS	9	0.0012	0.085	0.9898
LIPID CATABOLIC PROCESS	31	0.0013	0.025	0.5431
REGULATION OF MITOSIS	33	0.0013	0.025	0.5524
RESPONSE TO EXTERNAL STIMULUS	251	0.0014	0.020	0.4678
PHOSPHOINOSITIDE METABOLIC PROCESS	26	0.0014	0.072	0.9590
COFACTOR TRANSPORT	10	0.0014	0.150	1.0000
ANATOMICAL STRUCTURE FORMATION	46	0.0015	0.027	0.5996
CYTOPLASMIC PART	1086	0.0016	0.027	0.5827
REGULATION OF CELL ADHESION	32	0.0016	0.027	0.5917
GROWTH FACTOR BINDING	26	0.0016	0.037	0.7773
CYTOKINE ACTIVITY	86	0.0017	0.018	0.4184
VASCULATURE DEVELOPMENT	43	0.0018	0.028	0.6296
REGULATION OF CYTOSKELETON ORGANIZATION AND BIOGENESIS	26	0.0020	0.031	0.6966
NUCLEUS	1104	0.0021	0.028	0.6174
MICROTUBULE POLYMERIZATION OR DEPOLYMERIZATION	10	0.0021	0.183	1.0000
REGULATION OF CYTOKINE SECRETION	11	0.0022	0.113	0.9995
VOLTAGE GATED CHANNEL ACTIVITY	61	0.0023	0.034	0.7354
MICROTUBULE ASSOCIATED COMPLEX	40	0.0024	0.030	0.6764
POSITIVE REGULATION OF BIOLOGICAL PROCESS	577	0.0025	0.029	0.6608
DEFENSE RESPONSE	207	0.0026	0.029	0.6470
CYTOKINE BINDING	35	0.0028	0.032	0.7152
STRUCTURAL CONSTITUENT OF MUSCLE	27	0.0028	0.037	0.7781
NUCLEAR UBIQUITIN LIGASE COMPLEX	7	0.0029	0.203	1.0000
INTERLEUKIN 1 SECRETION	7	0.0032	0.117	0.9999
FATTY ACID BETA OXIDATION	9	0.0032	0.225	1.0000
CYTOSKELETON	309	0.0033	0.037	0.7816
CELLULAR PROTEIN METABOLIC PROCESS	884	0.0033	0.045	0.8391
ENZYME INHIBITOR ACTIVITY	87	0.0036	0.034	0.7409

Table 2 – continued from previous page

Pathway	♯ SNPs	p-value	FDR	FWER
HYDROLASE ACTIVITY HYDROLYZING O GLYCOSYL COMPOUNDS	24	0.0037	0.048	0.8629
RNA METABOLIC PROCESS	677	0.0044	0.060	0.9236
ANATOMICAL STRUCTURE MORPHOGENESIS	308	0.0045	0.049	0.8725
INTRINSIC TO MEMBRANE	1064	0.0046	0.066	0.9455
POSITIVE REGULATION OF SECRETION	16	0.0046	0.083	0.9863
CELLULAR MACROMOLECULE METABOLIC PROCESS	896	0.0047	0.058	0.9155
SERINE TYPE ENDOPEPTIDASE ACTIVITY	34	0.0048	0.056	0.9054
ISOPRENOID METABOLIC PROCESS	9	0.0049	0.201	1.0000
PROTEIN KINASE CASCADE	240	0.0050	0.072	0.9566
OXIDOREDUCTASE ACTIVITY GO 0016706	9	0.0051	0.206	1.0000
VOLTAGE GATED CATION CHANNEL ACTIVITY	56	0.0052	0.066	0.9438
FATTY ACID OXIDATION	16	0.0054	0.091	0.9936
INTERLEUKIN BINDING	18	0.0057	0.076	0.9741
PROTEIN POLYMERIZATION	17	0.0057	0.084	0.9873
NEGATIVE REGULATION OF CELL DIFFERENTIATION	22	0.0059	0.075	0.9747
POSITIVE REGULATION OF CYTOKINE SECRETION	7	0.0060	0.174	1.0000
HORMONE METABOLIC PROCESS	25	0.0061	0.075	0.9727
REGULATION OF METABOLIC PROCESS	640	0.0062	0.074	0.9679
CELL CELL ADHESION	77	0.0063	0.066	0.9395
RESPONSE TO CHEMICAL STIMULUS	258	0.0063	0.074	0.9694
REGULATION OF HEART CONTRACTION	21	0.0066	0.084	0.9894
PROTEIN KINASE BINDING	51	0.0067	0.076	0.9720
RNA EXPORT FROM NUCLEUS	17	0.0067	0.113	0.9993
CELL PROLIFERATION GO 0008283	408	0.0070	0.065	0.9398
INTRINSIC TO ORGANELLE MEMBRANE	43	0.0071	0.072	0.9630
CELLULAR COMPONENT ASSEMBLY	241	0.0073	0.079	0.9797
INTEGRAL TO MEMBRANE	1054	0.0073	0.083	0.9858
REGULATION OF MULTICELLULAR ORGANISMAL PROCESS	122	0.0074	0.066	0.9469
INTEGRAL TO ORGANELLE MEMBRANE	42	0.0074	0.072	0.9629
PROTEIN HETERODIMERIZATION ACTIVITY	69	0.0079	0.083	0.9866
NEUROGENESIS	78	0.0082	0.085	0.9891
ENDOTHELIAL CELL PROLIFERATION	11	0.0085	0.084	0.9881
REGULATION OF CELLULAR METABOLIC PROCESS	631	0.0087	0.084	0.9899
NEGATIVE REGULATION OF CELL PROLIFERATION	132	0.0090	0.082	0.9840
POSITIVE REGULATION OF PROTEIN SECRETION	8	0.0090	0.194	1.0000
SERINE HYDROLASE ACTIVITY	38	0.0093	0.091	0.9946
SERINE TYPE PEPTIDASE ACTIVITY	38	0.0093	0.091	0.9946
NEGATIVE REGULATION OF CELLULAR PROTEIN METABOLIC PROCESS	32	0.0093	0.098	0.9972
NEGATIVE REGULATION OF PROTEIN METABOLIC PROCESS	34	0.0095	0.099	0.9971
DNA MODIFICATION	10	0.0098	0.313	1.0000
BIOPOLYMER METABOLIC PROCESS	1350	0.0101	0.093	0.9945
RNA CATABOLIC PROCESS	20	0.0101	0.121	0.9999
KINESIN COMPLEX	11	0.0101	0.283	1.0000
INTERLEUKIN RECEPTOR ACTIVITY	16	0.0102	0.108	0.9991
HYDROLASE ACTIVITY ACTING ON ESTER BONDS	218	0.0104	0.094	0.9955
REGULATION OF CELLULAR COMPONENT ORGANIZATION AND BIOGENESIS	103	0.0107	0.092	0.9951
CALCIUM ION TRANSMEMBRANE TRANSPORTER ACTIVITY	9	0.0109	0.113	0.9995
ENDOPEPTIDASE ACTIVITY	95	0.0112	0.096	0.9961
GTPASE REGULATOR ACTIVITY	105	0.0112	0.099	0.9978
MICROTUBULE CYTOSKELETON ORGANIZATION AND BIOGENESIS	31	0.0116	0.115	0.9996
I KAPPAB KINASE NF KAPPAB CASCADE	94	0.0118	0.096	0.9960
POSITIVE REGULATION OF MULTICELLULAR ORGANISMAL PROCESS	52	0.0122	0.096	0.9960
INTRINSIC TO PLASMA MEMBRANE	788	0.0123	0.105	0.9984
CELLULAR MACROMOLECULE CATABOLIC PROCESS	88	0.0125	0.098	0.9971
AXONOGENESIS	34	0.0125	0.103	0.9982
MRNA METABOLIC PROCESS	62	0.0126	0.105	0.9989
BIOPOLYMER CATABOLIC PROCESS	97	0.0130	0.099	0.9978
DELAYED RECTIFIER POTASSIUM CHANNEL ACTIVITY	9	0.0131	0.112	0.9996
REGULATION OF BLOOD PRESSURE	16	0.0134	0.211	1.0000
NUCLEAR EXPORT	29	0.0135	0.098	0.9970
IMMUNE SYSTEM PROCESS	254	0.0135	0.098	0.9972
HEMATOPOIETIN INTERFERON CLASS D200 DOMAIN CYTOKINE RECEPTOR ACTIVITY	28	0.0136	0.104	0.9986
CELL SURFACE RECEPTOR LINKED SIGNAL TRANSDUCTION GO 0007166	505	0.0136	0.108	0.9991
T CELL DIFFERENTIATION	13	0.0136	0.244	1.0000
DNA DIRECTED RNA POLYMERASE II HOLOENZYME	50	0.0140	0.111	0.9993

Table 2 – continued from previous page

Pathway	♯ SNPs	p-value	FDR	FWER
HEMOPOIETIC OR LYMPHOID ORGAN DEVELOPMENT	64	0.0145	0.113	0.9995
CELLULAR MORPHOGENESIS DURING DIFFERENTIATION	40	0.0147	0.114	0.9996
POSITIVE REGULATION OF METABOLIC PROCESS	197	0.0148	0.112	0.9995
COENZYME BIOSYNTHETIC PROCESS	10	0.0149	0.235	1.0000
REGULATION OF ORGANELLE ORGANIZATION AND BIOGENESIS	34	0.0152	0.113	0.9993
INTEGRAL TO ENDOPLASMIC RETICULUM MEMBRANE	20	0.0152	0.115	0.9999
INTRINSIC TO ENDOPLASMIC RETICULUM MEMBRANE	20	0.0152	0.115	0.9999
REGULATION OF PROTEIN POLYMERIZATION	10	0.0153	0.262	1.0000
GENERATION OF NEURONS	70	0.0155	0.112	0.9993
GUANYL NUCLEOTIDE EXCHANGE FACTOR ACTIVITY	40	0.0155	0.113	0.9996
HEMOPOIESIS	63	0.0161	0.116	0.9999
GROWTH FACTOR ACTIVITY	46	0.0162	0.112	0.9997
IMMUNE SYSTEM DEVELOPMENT	67	0.0164	0.121	0.9999
MACROMOLECULE CATABOLIC PROCESS	112	0.0167	0.112	0.9996
REGULATION OF PROTEIN METABOLIC PROCESS	134	0.0170	0.114	0.9995
PHOSPHORIC ESTER HYDROLASE ACTIVITY	125	0.0173	0.121	0.9999
INTEGRAL TO PLASMA MEMBRANE	780	0.0173	0.125	1.0000
CYTOKINE SECRETION	13	0.0173	0.131	1.0000
REGULATION OF CYTOKINE PRODUCTION	21	0.0174	0.114	0.9996
RESPONSE TO DRUG	20	0.0186	0.129	1.0000
CYTOSOL	171	0.0190	0.115	0.9999
PROTEIN COMPLEX ASSEMBLY	141	0.0193	0.121	0.9999
REGULATION OF ENDOTHELIAL CELL PROLIFERATION	9	0.0195	0.119	0.9999
MACROMOLECULAR COMPLEX ASSEMBLY	225	0.0195	0.122	0.9999
REGULATION OF GENE EXPRESSION	542	0.0196	0.126	1.0000
HYDROLASE ACTIVITY ACTING ON GLYCOSYL BONDS	32	0.0198	0.132	1.0000
CELL DEVELOPMENT	468	0.0199	0.126	1.0000
REGULATION OF TRANSCRIPTION	459	0.0200	0.126	0.9999
GLYCEROPHOSPHOLIPID METABOLIC PROCESS	37	0.0201	0.124	0.9999
NEGATIVE REGULATION OF DEVELOPMENTAL PROCESS	160	0.0201	0.131	1.0000
ZINC ION BINDING	68	0.0204	0.125	0.9999
CELL SURFACE	64	0.0209	0.126	0.9999
RESPONSE TO WOUNDING	154	0.0217	0.125	1.0000
NEGATIVE REGULATION OF CYTOSKELETON ORGANIZATION AND BIOGENESIS	8	0.0218	0.354	1.0000
GOLGI MEMBRANE	41	0.0227	0.135	1.0000
PEPTIDE RECEPTOR ACTIVITY	40	0.0227	0.137	1.0000
ORGAN MORPHOGENESIS	118	0.0230	0.136	1.0000
CELL CELL SIGNALING	316	0.0233	0.134	1.0000
NEURON DIFFERENTIATION	64	0.0240	0.138	1.0000
GENERAL RNA POLYMERASE II TRANSCRIPTION FACTOR ACTIVITY	29	0.0240	0.162	1.0000
RESPONSE TO ABIOTIC STIMULUS	71	0.0241	0.131	1.0000
PROTEIN DIMERIZATION ACTIVITY	156	0.0242	0.140	1.0000
POSITIVE REGULATION OF CELLULAR METABOLIC PROCESS	193	0.0243	0.148	1.0000
REGULATION OF PROTEIN SECRETION	16	0.0243	0.157	1.0000
SUGAR BINDING	26	0.0249	0.237	1.0000
RESPONSE TO OTHER ORGANISM	62	0.0250	0.136	1.0000
DETECTION OF CHEMICAL STIMULUS	16	0.0251	0.157	1.0000
TRIACYLGLYCEROL METABOLIC PROCESS	9	0.0255	0.382	1.0000
RESPONSE TO BIOTIC STIMULUS	95	0.0263	0.136	1.0000
LYMPHOCYTE DIFFERENTIATION	22	0.0266	0.151	1.0000
MULTI ORGANISM PROCESS	126	0.0278	0.159	1.0000
MICROTUBULE CYTOSKELETON	132	0.0284	0.150	1.0000
PHOSPHOLIPID METABOLIC PROCESS	60	0.0285	0.159	1.0000
MRNA SPLICE SITE SELECTION	8	0.0287	0.396	1.0000
LEUKOCYTE DIFFERENTIATION	32	0.0296	0.160	1.0000
T CELL ACTIVATION	35	0.0297	0.160	1.0000
ENZYME REGULATOR ACTIVITY	261	0.0302	0.170	1.0000
TRANSCRIPTION	609	0.0303	0.164	1.0000
ENZYME LINKED RECEPTOR PROTEIN SIGNALING PATHWAY	121	0.0304	0.166	1.0000
PLASMA MEMBRANE PART	921	0.0312	0.169	1.0000
REGULATION OF MOLECULAR FUNCTION	257	0.0314	0.162	1.0000
LEUKOCYTE ACTIVATION	52	0.0318	0.163	1.0000
VOLTAGE GATED SODIUM CHANNEL ACTIVITY	9	0.0318	0.224	1.0000
INTRACELLULAR SIGNALING CASCADE	533	0.0319	0.162	1.0000
REGULATION OF INTRACELLULAR TRANSPORT	17	0.0319	0.207	1.0000
ANDROGEN RECEPTOR SIGNALING PATHWAY	8	0.0326	0.408	1.0000
GLYCOPROTEIN BIOSYNTHETIC PROCESS	53	0.0337	0.178	1.0000
TRANSITION METAL ION BINDING	84	0.0343	0.183	1.0000
ANTI APOPTOSIS	92	0.0345	0.188	1.0000

Table 2 – continued from previous page

Pathway	♯ SNPs	p-value	FDR	FWER
MICROVILLUS	11	0.0346	0.194	1.0000
PHOSPHORIC MONOESTER HYDROLASE ACTIVITY	91	0.0347	0.183	1.0000
PATTERN SPECIFICATION PROCESS	22	0.0364	0.202	1.0000
CORNIFIED ENVELOPE	13	0.0368	0.202	1.0000
TELOMERIC DNA BINDING	9	0.0369	0.383	1.0000
HUMORAL IMMUNE RESPONSE	24	0.0372	0.189	1.0000
ACTIN CYTOSKELETON	106	0.0381	0.188	1.0000
CYSTEINE TYPE PEPTIDASE ACTIVITY	47	0.0385	0.190	1.0000
CELL ACTIVATION	59	0.0387	0.192	1.0000
MEDIATOR COMPLEX	11	0.0387	0.310	1.0000
MEMBRANE PART	1317	0.0389	0.195	1.0000
REGULATION OF NUCLEOCYTOPLASMIC TRANSPORT	14	0.0390	0.226	1.0000
KINASE BINDING	58	0.0391	0.188	1.0000
ACTIVATION OF NF KAPPAB TRANSCRIPTION FACTOR	14	0.0394	0.207	1.0000
GTPASE ACTIVITY	77	0.0398	0.205	1.0000
GLAND DEVELOPMENT	10	0.0403	0.339	1.0000
RAS GUANYL NUCLEOTIDE EXCHANGE FACTOR ACTIVITY	16	0.0409	0.207	1.0000
PROTEIN BINDING BRIDGING	45	0.0415	0.203	1.0000
NEGATIVE REGULATION OF CATALYTIC ACTIVITY	51	0.0419	0.202	1.0000
ACUTE INFLAMMATORY RESPONSE	9	0.0426	0.418	1.0000
STEROID BIOSYNTHETIC PROCESS	16	0.0427	0.230	1.0000
SENSORY ORGAN DEVELOPMENT	10	0.0434	0.312	1.0000
COFACTOR BIOSYNTHETIC PROCESS	19	0.0437	0.230	1.0000
POSITIVE REGULATION OF RESPONSE TO STIMULUS	36	0.0438	0.207	1.0000
POSITIVE REGULATION OF PROTEIN METABOLIC PROCESS	60	0.0439	0.207	1.0000
ACETYLGALACTOSAMINYLTRANSFERASE ACTIVITY	10	0.0441	0.247	1.0000
PROTEIN CATABOLIC PROCESS	58	0.0442	0.207	1.0000
NUCLEAR TRANSPORT	73	0.0450	0.209	1.0000
NUCLEOCYTOPLASMIC TRANSPORT	73	0.0450	0.209	1.0000
HEMATOPOIETIN INTERFERON CLASS D200 DOMAIN CYTOKINE RECEPTOR BINDING	22	0.0450	0.378	1.0000
PHOTOTRANSDUCTION	11	0.0454	0.340	1.0000
TISSUE DEVELOPMENT	111	0.0456	0.207	1.0000
RECEPTOR SIGNALING PROTEIN ACTIVITY	66	0.0464	0.211	1.0000
NEURITE DEVELOPMENT	44	0.0466	0.219	1.0000
PATTERN BINDING	37	0.0467	0.211	1.0000
ACTIN FILAMENT POLYMERIZATION	13	0.0475	0.261	1.0000
NEGATIVE REGULATION OF TRANSLATION	16	0.0480	0.419	1.0000
CELL FRACTION	396	0.0482	0.218	1.0000
REGULATION OF CELL CYCLE	148	0.0485	0.224	1.0000
ORGAN DEVELOPMENT	454	0.0491	0.222	1.0000
NEGATIVE REGULATION OF HYDROLASE ACTIVITY	9	0.0498	0.417	1.0000
LIPID RAFT	23	0.0499	0.226	1.0000

Table 3 - Pathways in MSigDB C5 collection identified by the two methods with p-value $P \leq 0.05$ and the corresponding FDR, in alphabetical order

Table 3: Pathways in MSigDB C5 collection identified by the two methods with p-value ≤ 0.05 and the corresponding FDR, in alphabetical order

			RS-SNP		GSEA-SNP	
Pathway	♯ genes	♯ SNPs	p-value	FDR	p-value	FDR
ACETYLGALACTOSAMINYLTRANSFER ACTIVITY	11	201	0.0453	0.3146	0.0441	0.247
ACTIVATION OF NF KAPPAB TRANSCRIPTION FACTOR	16	188	0.0290	0.2983	0.0394	0.207
ANATOMICAL STRUCTURE MORPHOGENESIS	326	4717	0.0147	0.2762	0.0045	0.049
AXONOGENESIS	36	1218	0.0026	0.1487	0.0125	0.103
CELL DEVELOPMENT	491	7007	0.0206	0.2993	0.0199	0.126
CELLULAR MACROMOLECULE METABOLIC PROCESS	967	11400	0.0304	0.3415	0.0047	0.058
CELLULAR MORPHOGENESIS DURING DIFFERENTIATION	42	1280	0.0029	0.1591	0.0147	0.114
CELLULAR PROTEIN METABOLIC PROCESS	954	11291	0.0266	0.3297	0.0033	0.045
COFACTOR TRANSPORT	10	68	0.0287	0.2909	0.0014	0.150
COFACTOR TRANSPORTER ACTIVITY	9	60	0.0256	0.2668	0.0007	0.160
CYSTEINE TYPE PEPTIDASE ACTIVITY	51	310	0.0393	0.3779	0.0385	0.190
CYTOKINE ACTIVITY	90	478	0.0044	0.2031	0.0017	0.018

Table 3 – continued from previous page

			RS-SNP		GSEA-SNP	
CYTOKINE BINDING	38	262	0.0014	0.1797	0.0028	0.032
CYTOKINE SECRETION	15	162	0.0405	0.3375	0.0173	0.131
CYTOSKELETON	333	4130	0.0147	0.2630	0.0033	0.037
CYTOSOL	181	1826	0.0091	0.2424	0.0190	0.115
DEFENSE RESPONSE	230	1620	0.0166	0.2959	0.0026	0.029
DEFENSE RESPONSE TO BACTERIUM	21	144	0.0224	0.2698	0.0004	0.028
DETECTION OF BIOTIC STIMULUS	10	67	0.0135	0.2402	0.0000	0.125
DETECTION OF EXTERNAL STIMULUS	22	145	0.0107	0.2393	0.0000	0.007
ENZYME BINDING	158	2083	0.0029	0.1780	0.0006	0.011
ENZYME INHIBITOR ACTIVITY	96	698	0.0026	0.2353	0.0036	0.034
ENZYME REGULATOR ACTIVITY	282	3866	0.0015	0.2099	0.0302	0.170
GENERATION OF NEURONS	74	2023	0.0020	0.1687	0.0155	0.112
GTPASE ACTIVITY	82	666	0.0280	0.2961	0.0398	0.205
GTPASE REGULATOR ACTIVITY	111	2514	0.0105	0.2486	0.0112	0.099
GUANYL NUCLEOTIDE EXCHANGE FACTOR ACTIVITY	42	1177	0.0090	0.2476	0.0155	0.113
HEMATOPOIETIN INTERFERON CLASS D200 DOMAIN CYTOKINE RECEPTOR ACTIVITY	30	317	0.0025	0.1912	0.0136	0.104
HEMATOPOIETIN INTERFERON CLASS D200 DOMAIN CYTOKINE RECEPTOR BINDING	22	72	0.0270	0.3060	0.0450	0.378
HYDROLASE ACTIVITY ACTING ON GLYCOSYL BONDS	34	424	0.0044	0.08034	0.0198	0.132
HYDROLASE ACTIVITY HYDROLYZING O GLYCOSYL COMPOUNDS	25	369	0.0040	0.06187	0.0037	0.048
I KAPPAB KINASE NF KAPPAB CASCADE	101	666	0.0228	0.3158	0.0118	0.096
IMMUNE SYSTEM PROCESS	277	2492	0.0120	0.2631	0.0135	0.098
INTEGRAL TO MEMBRANE	1127	16058	0.0092	0.2687	0.0073	0.083
INTEGRAL TO PLASMA MEMBRANE	833	12627	0.0033	0.2499	0.0173	0.125
INTERLEUKIN 1 SECRETION	8	95	0.0148	0.2490	0.0032	0.117
INTERLEUKIN BINDING	21	158	0.0013	0.1666	0.0057	0.076
INTERLEUKIN RECEPTOR ACTIVITY	18	143	0.0011	0.1543	0.0102	0.108
INTRINSIC TO MEMBRANE	1137	16350	0.0058	0.26102	0.0046	0.066
INTRINSIC TO PLASMA MEMBRANE	841	12909	0.0024	0.2465	0.0123	0.105
KINESIN COMPLEX	12	74	0.0294	0.3144	0.0101	0.283
MEMBRANE	1683	25238	0.0002	0.2098	0.0002	0.003
MEMBRANE PART	1413	20710	0.0154	0.3162	0.0389	0.195
MICROTUBULE ASSOCIATED COMPLEX	44	417	0.0063	0.0747	0.0024	0.030
MICROTUBULE CYTOSKELETON	143	1179	0.0078	0.1793	0.0284	0.150
MICROTUBULE CYTOSKELETON ORGANIZATION AND BIOGENESIS	32	209	0.0071	0.0531	0.0116	0.115
MICROTUBULE POLYMERIZATION OR DEPOLYMERIZATION	10	101	0.0083	0.0523	0.0021	0.183
MULTI ORGANISM PROCESS	142	868	0.0026	0.2381	0.0278	0.159
NEURITE DEVELOPMENT	46	1268	0.0027	0.1521	0.0466	0.219
NEUROGENESIS	82	2099	0.0025	0.1665	0.0082	0.085
NEURON DIFFERENTIATION	68	1952	0.0025	0.1572	0.0240	0.138
NUCLEAR UBIQUITIN LIGASE COMPLEX	8	79	0.0049	0.1370	0.0029	0.203
NUCLEUS	1215	10615	0.0042	0.2484	0.0021	0.028
PEPTIDASE ACTIVITY	157	1482	0.0336	0.3280	0.0000	0.002
PLASMA MEMBRANE	1213	20009	0.0013	0.24644	0.0000	0.001
PLASMA MEMBRANE PART	987	16216	0.0088	0.26892	0.0312	0.169
POSITIVE REGULATION OF CELLULAR METABOLIC PROCESS	208	2503	0.0438	0.3697	0.0243	0.148
POSITIVE REGULATION OF CELLULAR PROCESS	585	6716	0.0438	0.3763	0.0012	0.014
POSITIVE REGULATION OF CYTOKINE PRODUCTION	14	103	0.0012	0.1594	0.0009	0.072
POSITIVE REGULATION OF CYTOKINE SECRETION	7	89	0.0260	0.2503	0.006	0.174
POSITIVE REGULATION OF METABOLIC PROCESS	214	2529	0.0268	0.3185	0.0148	0.112
POSITIVE REGULATION OF MULTICELLULAR ORGANISMAL PROCESS	58	444	0.0250	0.3126	0.0122	0.096
POSITIVE REGULATION OF PROTEIN METABOLIC PROCESS	65	474	0.0014	0.06471	0.0439	0.207
POSITIVE REGULATION OF PROTEIN SECRETION	8	94	0.0270	0.2658	0.009	0.194
POSITIVE REGULATION OF SECRETION	16	128	0.0366	0.3225	0.0046	0.083
PROTEIN KINASE BINDING	52	494	0.0497	0.3713	0.0067	0.076
PROTEIN METABOLIC PROCESS	1052	12645	0.0103	0.2669	0.0001	0.006
PROTEIN POLYMERIZATION	17	241	0.0093	0.06268	0.0057	0.084
RAS GUANYL NUCLEOTIDE EXCHANGE FACTOR ACTIVITY	17	550	0.0073	0.2467	0.0409	0.207
RECEPTOR ACTIVITY	498	8905	0.0296	0.3497	0.0000	0.000

Table 3 – continued from previous page

			RS-SNP		GSEA-SNP	
RECEPTOR BINDING	315	3004	0.0020	0.2381	0.0003	0.002
REGULATION OF BLOOD PRESSURE	18	99	0.0261	0.26736	0.0134	0.211
REGULATION OF CATABOLIC PROCESS	15	79	0.0319	0.3256	0.0001	0.074
REGULATION OF CELLULAR COMPONENT ORGANIZATION AND BIOGENESIS	108	1529	0.0014	0.1676	0.0107	0.092
REGULATION OF CELLULAR METABOLIC PROCESS	684	6937	0.0329	0.3453	0.0087	0.084
REGULATION OF CYTOKINE PRODUCTION	23	185	0.0051	0.2523	0.0174	0.114
REGULATION OF CYTOKINE SECRETION	13	102	0.0302	0.2676	0.0022	0.113
REGULATION OF CYTOSKELETON ORGANIZATION AND BIOGENESIS	26	345	0.0014	0.0626	0.0020	0.031
REGULATION OF METABOLIC PROCESS	694	6998	0.0228	0.3236	0.0062	0.074
REGULATION OF ORGANELLE ORGANIZATION AND BIOGENESIS	35	397	0.0019	0.0647	0.0152	0.113
REGULATION OF PROTEIN METABOLIC PROCESS	145	1017	0.0028	0.1541	0.0170	0.114
REGULATION OF PROTEIN POLYMERIZATION	10	127	0.0089	0.06945	0.0153	0.262
REGULATION OF PROTEIN SECRETION	18	179	0.0051	0.1766	0.0243	0.157
RESPONSE TO BACTERIUM	26	163	0.0199	0.2711	0.0001	0.014
RESPONSE TO BIOTIC STIMULUS	105	560	0.0114	0.2496	0.0263	0.136
RESPONSE TO EXTERNAL STIMULUS	269	2798	0.0368	0.3682	0.0014	0.020
RESPONSE TO OTHER ORGANISM	72	367	0.0118	0.2645	0.0250	0.136
STRUCTURAL CONSTITUENT OF MUSCLE	30	437	0.0109	0.2484	0.0028	0.037
TRANSMEMBRANE RECEPTOR ACTIVITY	353	6830	0.0224	0.3238	0.0000	0.001
