# Supplementary material for: RS-SNP: a random-set method for genome-wide association studies
Source: BMC Genomics. 2011 Mar 30;12:166. doi: 10.1186/1471-2164-12-166 (PMC3079664; doi:10.1186/1471-2164-12-166)
Supplement: Additional file 3 — RS-SNP package. The proposed RS-SNP software is contained in this compressed file, together with: • the help documentation, • example files with the SNP-gene mapping and gene-pathway mapping; • example of input files. [file 1471-2164-12-166-S3.ZIP › daddabbo_bmc_genomics_af3_rev1/help_doc/compute_rs.pdf]

Using compute_rs.m for pathway-based association analysis on GWAS

The compute_rs.m program is designed to perform pathway-based genome-wide association (GWA) tests on high-density SNP genotyping data.

1. Introduction
2. General overview of the procedure
3. Input files
    1. Association results file
    2. Association permutation results file
    3. SNP-gene mapping file
    4. Pathway definition file
4. Testing procedure
    1. Testing individual association permutation results file
    2. Combining several mat files together to recalculate enrichment statistics for pathways
    3. Tweaking parameters
    4. Other functionalities of the program

Introduction

This program takes GWA association results (chi2 and P values for all markers for each phenotype permutation), a SNP-to-gene mapping file, and a pathway annotation file to perform pathway-based association tests. The algorithm belongs to the class of Random Set methods (Efron, Tibshirani, On testing the significance of sets of genes. *The Annals of Applied Statistics*, **1**(1), pp.107–129, 2007) with certain modifications.

Briefly, the method assesses whether the number y of SNPs with p-value $P \leq \alpha$ belonging to a given SNP set S is statistically significant. This method simply assigns all SNPs near a gene to the gene, the genes to a given gene set and use phenotype-based permutation for adjusting statistical significance. The most significantly associated pathways can be identified from a set of candidate pathways, together with statistical significance calculated from permutation procedure.

General overview of the procedure

A general overview of the testing procedure used in this program is given in the following figure. This program uses permutation test extensively, so the entire procedure is computationally expensive. Generally speaking, it is possible to run different times compute_rs.m and then combine the results by using the combine_rs.m program to generate the pathway significance file.

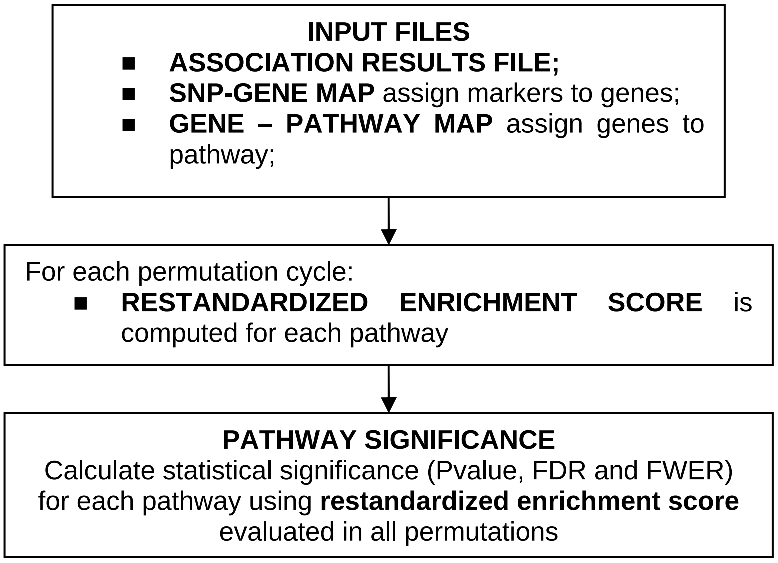

Input files

The program requires three main input files:

1. association results file (containing also the association permutation results);
2. SNP-gene mapping file;
3. pathway definition file.

Their formats are briefly described below.

Association results file

The association results file contains chi2 values and P-values for genome-wide association tests on all markers to be tested in pathway-based approach. It has a simple six-columns tab-separated format: the first four columns are marker-id, chromosome, position and alleles; the fifth column is the chi2 value; the sixth column is the P value.

If you happen to use compute_association.m program to calculate the chi2 and P-values, its output file named “gwa_results.txt” can be used as input file for compute_rs.m.

Association permutation results file

The permutation results file contains test statistic values for all SNPs for all permutation cycles, with one marker per line. The first line of the permutation file is called header line.

To explain this in more detail, see the permutation results file below:

```
Marker name    Chr     Position       A:B     trend chi2     trend P Perm chi2      Perm P
rs3677638      1       158297960      A:B     2       0.157299       ,2,2,8,0,2,0,2,0,0,0
       ,0.157299,0.157299,0.00467773,1,0.157299,1,0.157299,1,1,1
rs3685643      1       164086737      A:B     0.258065       0.611453
       ,2.32258,2.32258,0.258065,0.258065,2.32258,0.258065,0.258065,2.32258,2.32258,2.32258
       ,0.127508,0.127508,0.611453,0.611453,0.127508,0.611453,0.611453,0.127508,0.127508,0.127508
```

```
rs13476259	1	179106265	A:B	1.14286	0.285049
	,1.14286,1.14286,1.14286,1.14286,1.14286,1.14286,1.14286,1.14286,1.14286,1.14286
	,0.285049,0.285049,0.285049,0.285049,0.285049,0.285049,0.285049,0.285049,0.285049,0.285049
mAV22849619	1	188585535	A:B	1.14286	0.285049
	,1.14286,1.14286,1.14286,1.14286,1.14286,1.14286,1.14286,1.14286,1.14286,1.14286
	,0.285049,0.285049,0.285049,0.285049,0.285049,0.285049,0.285049,0.285049,0.285049,0.285049
rs3689947	1	194202719	A:B	0	1
	,0,2.66667,2.66667,0,2.66667,0,0,2.66667,2.66667,0
	,1,0.10247,0.10247,1,0.10247,1,1,0.10247,0.10247,1
rs13476331	2	16018358	A:B	1.14286	0.285049
	,1.14286,1.14286,1.14286,1.14286,1.14286,1.14286,1.14286,1.14286,1.14286,1.14286
	,0.285049,0.285049,0.285049,0.285049,0.285049,0.285049,0.285049,0.285049,0.285049,0.285049
AEL-2_23847726	2	23709712	A:B	0	1
	,2.66667,2.66667,0,2.66667,2.66667,2.66667,0,2.66667,2.66667,2.66667
	,0.10247,0.10247,1,0.10247,0.10247,0.10247,1,0.10247,0.10247,0.10247
rs6295520	2	42896745	A:B	0	1	,1,1,1,0,1,4,0,1,1,1
	,0.317311,0.317311,0.317311,1,0.317311,0.0455003,1,0.317311,0.317311,0.317311
rs13476507	2	54627980	A:B	0.205128	0.650613
	,0.205128,0.205128,1.84615,0.205128,0.205128,5.12821,0.205128,0.205128,0.205128,0.205128
	,0.650613,0.650613,0.174231,0.650613,0.650613,0.0235401,0.650613,0.650613,0.650613,0.650613
```

Each line contains 8 tab-delimited columns, and the first four columns are general description of the SNP marker, while the rest columns are statistical information on the marker in the data set. The compute_rs.m program uses in its analysis the content of “chi2” and “Perm chi2” columns (the fifth and the seventh columns) in every line, automatically excluding the header line. The “Perm chi2” column contains 10 numbers separated by comma, representing 10 chi2 values generated from 10 permutation cycles.

If you use other GWA program to perform the association tests, you have to reformat all the association results into the above file format.

SNP-gene mapping file

The SNP-gene mapping file is another tab-delimited file, with three columns per line: gene name, gene description, name list of SNPs that belonging to it. A SNP-gene mapping file drawn by the Affymetrix annotation files Mapping 250K_Nsp Annotations and Mapping 250K_Sty Annotations, CSV format, version 26, is here provided, assigning SNPs to each gene within a distance of 5kb from it (5kb upstream and 5kb downstream). If other SNP-gene distances have to be considered, an opportune SNP-gene mapping file has to be generated.

Pathway definition file

The pathway definition file is a tab-delimited file, with one pathway per line. The first two columns are the pathway id, pathway description, while the following columns are gene identifiers within the pathway. An example pathway file is shown below. This file contains two pathways, named GO0015884 and GO0007447, respectively. In this example, the pathway description and the pathway name coincides. The first pathway is composed of 29 genes, the second one of 4 genes.

```
GO0015884	GO0015884	FLOT1	FOLR	FOLR1	FOLR1_HUMAN	FOLR2	FOLR2_HUMAN
FOLR3	FOLR3_HUMAN	GP36	MFT	MFTC	MFTC_HUMAN	P14207	P14207_HUMAN	P15328
P15328_HUMAN	P41439	P41439_HUMAN	P41440	P41440_HUMAN	PDPN	PDPN_HUMAN
PSEC0003	PSEC0025	Q86YL7	Q86YL7_HUMAN	Q9H2D1	Q9H2D1_HUMAN	RFC1
S19A1_HUMAN	SLC19A1	SLC25A32
GO0007447	GO0007447	DVL2	DVL2_HUMAN	O14641	O14641_HUMAN
```

Some pathway definition files, drawn from MSigDB database, available at http://www.broad.mit.edu/gsea/msigdb/collections.jsp, are here provided. The MSigDB database is a useful source of pathway collections. The C2 and C5 gene collections are

probably most relevant for genome-wide association studies. (Note that the C5 collection is also a Gene Ontology collection)

You can also test your own candidate pathway, by making a simple tab-delimited file: note that the first two columns are pathway ID and pathway description, respectively, and all other columns are regarded as gene names.

Testing procedure

Testing individual permutation results files

Note that the example below assumed an association results file named “gwa_results.txt”, a snp-gene-mapping file and a gene set file called respectively “snp_gene_mapping.gmt” and “pathway.gmt”.

For example, the following command:

```
>> compute_rs('gwa_resuts.txt', 'snp_gene_mapping.gmt', 'pathway.gmt')
```

specifies only the name of input files. The first three input arguments are always strictly required; the other three parameters, if not specified, are set to their default values.

If the facultative input variables have to be specified they have to be introduced in the following order: rs_parameter_th, combine_flag, stat_flag.

The rs_parameter_th variable allows to set the p-value threshold useful to the method to consider markers associated to pathology at hand.

The combine_flag variable can be set equal to 0 if there are not gwa_results files to combine. Otherwise, it is set to the number of gwa results file to analyse.

The stat_flag variable has the following possible values: ‘full_genotypic’,’allelic’,’dominant’, ‘recessive’, ‘trend’. Note that this flag has to be set if and only if the input “gwa_results.txt” file contains results of all the associative tests.

The default value of facultative input variables are: rs_parameter_th = 0.01, combine_flag = 0 (i.e. there are no files to combine), stat_flag = ‘trend’.

An example of the output file, named “RS_analysis_results.txt” , is shown below:

```
Total_number_of_SNP = 95
Number_of_associated_SNP = 2
Pathway name   pathway size   stat value     stat value stand      pvalue  FDR     FWER
Pathway_1      49      0       -1.46743       0.61    0.61    0.61
Pathway_2      49      1       -0.0449212     0.46    0.5075  0.6
Pathway_3      49      2       1.37758 0.04   0.08    0.11
Pathway_4      49      2       1.37758 0.04   0.08    0.11
Pathway_5      49      2       1.37758 0.03   0.08    0.11
```

During the run of the program, the association results file is read together with the permutation statistic values for all the SNPs. Then, the snp-gene-mapping file and the gene set file are read. Each of the permutation cycle is analyzed and restandardized

statistical value is calculated for each pathway. The P-value, the FDR value and the FWER P-value for each pathway is written in the same line.

Combining several mat files together to recalculate enrichment statistics for pathways

As mentioned before, performing 1000 permutations for GWA is very time-consuming; therefore, it is generally recommended to split your job into 10 parts, each with 100 permutations. We can run the compute_rs.m program on each part separately, each time generating a .mat file. The combine_rs.m program merges the output files produced by compute_rs.m.

Suppose 10 permutation results files (each containing statistics for 100 permutations) are analyzed by the compute_rs.m program, and 10 mat files are generated by each run. To combine the results from all 1000 permutations, one can simply do:

```
>> combine_rs(10)
```

So all 10 mat files will be jointly analyzed, and final pathway significance values will be calculated.

Tweaking parameters

Multiple parameters and files can be tweaked in the program, and they may generate quite different enrichment results. Below we briefly describe several changes that can be made in the association calculation.

First, the testing strategy can be changed for case-control studies. The default association test is the linear trend test in the compute_association.m program, but another association test may be considered. If you use compute_association.m program for case-control studies, then a different testing strategy can be specified.

Next, the SNP-gene mapping file can be changed and the SNP-gene distance can be changed.

Finally, the pathway annotation file can be supplemented or even replaced.

Other functionality of the program

In the absence of a permutation association results file, the compute_rs.m program can apply genotype-based permutation, that is, it permutes the P-values and chi2 values between SNPs. This is not really recommended for SNP arrays with high marker-marker linkage disequilibrium (LD) patterns, as such permutation disrupts the LD structure and may generate biased results.
