# Supplementary material for: RS-SNP: a random-set method for genome-wide association studies
Source: BMC Genomics. 2011 Mar 30;12:166. doi: 10.1186/1471-2164-12-166 (PMC3079664; doi:10.1186/1471-2164-12-166)
Supplement: Additional file 3 — RS-SNP package. The proposed RS-SNP software is contained in this compressed file, together with: • the help documentation, • example files with the SNP-gene mapping and gene-pathway mapping; • example of input files. [file 1471-2164-12-166-S3.ZIP › daddabbo_bmc_genomics_af3_rev1/help_doc/compute_association.doc]

# Using compute_association.m for genome-wide association analysis

The ‘compute_association.m’ program in the RS-SNP package is written to perform simple single-marker association tests on data sets from genome-wide association studies (GWAS).

1. Introduction
2. General overview of the procedure
3. Input and output files
   1. Sample header file
   2. GT file
   3. Removed sample list file
   4. Removed marker list file
   5. Case-control association test result file
4. Case-control association test
   1. Simple usage
   2. Permutation procedure
   3. Changing individual and marker exclusion criteria
5. Advanced topics and notes
   1. Chromosome X processing
   2. Parallel computation

**Introduction**

The ‘compute_association.m’ program performs case-control analysis. The main features of the software are the following ones:

1. The program is written in Matlab, it is very powerful and the source code is easily modifiable by other users.
2. The program uses a GT file that contains one marker per line and one individual per column. This indicates that it is naturally capable of processing genotype calls generated from high-density SNP genotyping platforms without complicated file format conversion (which usually involves matrix transposition), and it is capable of analyzing data set that is already processed by the PLINK software with little effort in format conversion.
3. The program generates rich annotation information in outputs to facilitate biological interpretation of association results.
4. It implements a permutation scheme to calculate P-values on permutated data sets, and these permuted test statistics can be coupled with the ‘compute_rs.m’ program for pathway-based association tests on GWAS.
5. Finally, it is capable of performing association tests on millions of markers upon 1 million individuals.

However, the functionality of the program is restricted to simple single-marker association tests. It is unable to offer more complicated testing options (such as population structure inference, multi-marker tests) available from other software.

**General overview of the procedure**

**INPUT FILES**

Sample_header file & GT file

**QUALITY CONTROLS**

**(Exclusion criteria)**

All markers are scanned and for each sample is computed:

Call rate

Each marker is processed sequentially computing:

Call rate

MAF

HWE

**GENERATED OUTPUT FILES**

Removed sample list file & removed marker list file

**GWA ANALYSIS**

Each marker is processed sequentially with the exclusion of:

samples in the “Removed sample list file”

markers in the “Removed marker list file”

**OUTPUT FILE WITH FINAL RESULTS**

A general overview of the procedure used in this program is given in the above figure.

Unlike other GWA software that read genotype data for all individuals into memory as a large matrix, the ‘compute_association.m’ program scans the genotype file and process each line (each marker) sequentially. It performs automatically two step: in the first one individuals are submitted to quality controls and eventually removed; in the second one each marker is submitted to quality controls and it is either removed or analyzed for association.

**Input and output files**

The program requires two main input files, a sample header file and a genotype (GT) file. These data formats are briefly described below:

### Sample header file

The sample header file is a tab-delimited text file that records information in one line for each individual. The first three columns of each line are individual id, sex (male=1; female=2) and affection status (healthy control=1; affected patient=2). If different codes for affection status are used, the corresponding samples will not be considered in the analysis. Moreover, if different codes for sex are used and a marker on X chromosome is considered the corresponding sample will not be included in the analysis.

An example of a sample_header.txt file is shown below:

| sample1 | 1 | 2 |  |
| --- | --- | --- | --- |
| sample2 | 1 | 1 |  |
| sample3 | 2 | 1 |  |
| sample4 | 1 | 1 |  |
| sample5 | 2 | 2 |  |
| sample6 | 2 | 1 |  |
| sample7 | 2 | 1 |  |

Many more additional columns can be added in the file following the first 3 columns.

**GT file**

The GT file is a genotype file that contains information for one marker per line. In a canonical GT file, the first three columns are marker-id, chromosome and position, while the following columns contain the genotypes for each individual.

To explain this in more detail, see the first 10 lines of a canonical GT file below:

```
Name    Chr     Position        sample1        sample2        sample3        sample4        sample5        sample6        sample7
 rs10013542      4       60194938        BB      BB      BB      BB      BB      BB      BB
 rs10013547      4       175988420       BB      BB      BB      BB      BB      BB      BB
 rs10013571      4       28821638        AA      AA      AA      AA      AB      AA      AB
 rs10013576      4       27228651        AB      BB      AB      AB      BB      BB      BB
 rs10013588      4       132717336       AA      AA      AA      AA      AA      AA      AA
 rs10013604      4       43295922        AA      AB      AB      AB      AA      AA      AA
 rs1001362       16      55232359        BB      BB      AB      AB      AB      BB      BB
 rs10013632      4       117373068       AA      AA      AA      AA      AA      AA      AA
 rs10013649      4       162694792       AA      AA      AA      AA      AA      AA      AA
 rs10013727      4       142935271       AB      AB      BB      AB      BB      BB      BB
```

Each line contains 10 tab-delimited columns, and the first three columns are general descriptions of the SNP markers, while the remaining columns are the genotypes of the markers for each sample. Genotype calls can be written considering the actual alleles (A, C, G, T) or simply A and B. Missing genotypes can be specified as NC, NN, --, 00 or 0. Note that it is allowed to have one space between the two alleles in a genotype call (so “AB” and “A B” are both valid syntax for genotype calls).

**Removed sample list file**

The removed sample list file specifies a group of individuals to be excluded from analysis. This is a simple file format: each line contains the individual identifier, sex, affection status and missing genotype call rate value. If there are no sample to remove, the output file ‘removed_sample_list.txt’ is not generated.

**Removed marker list file**

The removed marker list file is used to specify a group of markers to be excluded from analysis. This is a simple file format: each line contains the removed marker name, the removed marker code (i.e. the reason of removal) and its corresponding value. The codes of marker removal are:

- ‘chr Y’ if the marker is on Y chromosome;
- 'call_rate' if the genotyping call rate is under a given threshold;
- 'allele_number' if a marker is not biallelic (to control for more than 2 alleles);
- 'maf' if the minor allele frequency is under a given threshold;
- 'hwe' if the p-value of exact test for Hardy-Weinberg equilibrium is under a given threshold.

**Case-control association test result file**

Case-control association test compares genotypes between two phenotype groups (case group and control group). Five different models are implemented to test the association of binary phenotypes with bi-allelic genotypes, including genotypic association test (2df χ^2^ test), allelic association test, Cochran-Armitage trend association test, dominant model association test and recessive model association test.

If all five tests are performed, the value of statistic and P values for all five tests are written in the output file, one marker per line.

If the permutation procedure is performed only one test is computed (by default the Cochran-Armitage trend association test) and the case-control association test result file contains the χ^2^ and P values and two additional columns (named chi2_perm and chi2_P_perm) with the values comma separated obtained in the permutation of labels.

**Case-control association test**

**Simple usage**

Note that all the examples below assumed a sample header file and a GT file called respectively sample_header.txt and gt.txt.

For example, the following command:

>> compute_association(‘sample_header.txt’, ‘gt.txt’)

specifies only the name of input files. The first two input arguments are always strictly required; the other seven parameters, if not specified, are set to their default values.

If the facultative input variables have to be specified they have to be introduced in the following order: ‘perm_flag’, ‘number_rand_perm’, ‘stat_flag’, ‘sample_nocall_rate_th’, ‘marker_nocall_rate_th’, ‘MAF_th’, ‘HWE_th’.

The ‘perm_flag’ variable can be set equal to 1 if the permutation procedure has to be performed. If it is set to any other value, the permutation procedure is not performed.

The ‘number_rand_perm’ variable allows to set the number of label random permutations performed.

The ‘stat_flag’ variable has the following possible value: ‘full_genotypic’,’allelic’,’dominant’, ‘recessive’, ‘trend’ or ‘all’ if all the associative tests have to be performed.

The ‘sample_nocall_rate_th’, ‘marker_nocall_rate_th’, ‘MAF_th’, ‘HWE_th’ variables allow to set suitable values for any threshold.

The default values of facultative input variables are: perm_flag = 1 (i.e. the permutation procedure is performed), number_rand_perm = 100, stat_flag = ‘trend’, sample_nocall_rate_th = 0.1, marker_nocall_rate_th = 0.1, MAF_th = 0.01, HWE_th = 0.001.

**Permutation procedure**

For case-control studies, the permutation procedure randomly flips the case-control status for all samples maintaining the same number of cases and controls in the permutated samples as in the original ones. Then, for each permutation, the GWA tests on all markers are performed. This procedure is particularly useful when one want to use pathway-based GWA analysis by the compute_rs.m program. The resulting output file contains two additional columns (chi2_perm and chi2_P_perm) that are read by the compute_rs.m program for testing candidate pathways. If the permutation procedure is performed and the statistical test is not specified by using the ‘stat_flag’ input variable, the Cochran-Armitage trend test is computed by default.

An example of output file (using 10 permutation cycles) is shown below:

```
Marker name	Chr	Position	A:B	trend chi2	trend P	Perm chi2	Perm P
rs3677638	1	158297960	A:B	2.5156	0.112724	,2.5156,4.32909,0.236247,0.0611523,0.376948,0.236247,0.0142519,0.0611523,1.81523,0.236247	,0.112724,0.0374664,0.62693,0.804684,0.539241,0.62693,0.904973,0.804684,0.177883,0.62693
rs3685643	1	164086737	A:B	0.837989	0.359973	,0.837989,0.301676,1.49314,0.837989,0.837989,2.52341,0.0335196,0.373286,0.301676,1.64246	,0.359973,0.582834,0.221729,0.359973,0.359973,0.112168,0.854733,0.541219,0.582834,0.199989
rs13476259	1	179106265	A:B	4.4618	0.034661	,0.959902,0.0427395,0.907791,2.97835,0.0323172,1.79369,1.79369,0.351935,0.0427395,1.79369	,0.327212,0.836217,0.340701,0.084385,0.857333,0.180478,0.180478,0.55302,0.836217,0.180478
mAV22849619	1	188585535	A:B	1.12692	0.288434	,0.00979296,0.00979296,0.236002,0.761504,0.761504,1.5863,0.00979296,2.09788,1.5863,0.761504	,0.921171,0.921171,0.627109,0.382858,0.382858,0.207856,0.921171,0.147504,0.207856,0.382858
rs3689947	1	194202719	A:B	3.18068	0.0745139	,0.458825,1.09424,0.0952791,1.09424,1.35979,1.35979,0.635856,2.00152,0.00360237,1.09424	,0.498174,0.295534,0.75757,0.295534,0.243575,0.243575,0.425215,0.157141,0.95214,0.295534
```

To perform 10 phenotype permutations and to obtain the above reported output file the following command line has been used:

>> compute_association(‘sample_header.txt’, ‘gt.txt’,1,10)

As there are five different models to perform association tests, the default permutated chi2 and P values are calculated using the Cochran-Armitage trend association test. If one wishes to use a different testing strategy in calculating chi2 and P values, one has to set the ‘stat_flag’ parameter in the command line. For example, if the allelic association test has to be computed, the command line is:

>> compute_association(‘sample_header.txt’, ‘gt.txt’,1,10,’allelic’)

The output of ‘compute_association.m’ can be fed into the ‘compute_rs.m’ program to calculate most significant pathways. See the documentation for ‘compute_rs.m’ program for more details.

When the permutation procedure is not desired the command line has to be:

>> compute_association(‘sample_header.txt’, ‘gt.txt’,0)

In this case all the association tests are computed by default and the following output file is obtained:

```
Marker name	Chr	Position	A:B	full_genotypic chi2	full_genotypic P	 allelic chi2	allelic P	dominant chi2	dominant P	recessive chi2	recessive P	trend chi2	trend P
rs3677638	1	158297960	A:B	4.33909	0.11423	3.96549	0.0464421	3.93887	0.0471823	0.207028	0.487607	2.5156	0.112724
rs3685643	1	164086737	A:B	0.897927	0.63829	1.22449	0.268481	0.864989	0.352346	0.213977	0.733718	0.837989	0.359973
rs13476259	1	179106265	A:B	7.96438	0.0186447	5.58694	0.0180948	0.0056756	0.00972499	0.199634	0.509788	4.4618	0.034661
mAV22849619	1	188585535	A:B	1.21073	0.545875	1.89884	0.168208	0.146505	0.331889	0.216108	0.456553	1.12692	0.288434
rs3689947	1	194202719	A:B	5.82434	0.0543577	4.87203	0.0272953	5.22518	0.0222621	0.207028	0.487607	3.18068	0.0745139
```

As we can see from the association results, for each marker, the statistic values and P values for five models are calculated. If only one association test has to be computed ( for example the allelic test) without label permutation the command line is:

>> compute _association(‘sample_header.txt’, ‘gt.txt’,0,0,’allelic’)

Some considerations are in order:

1) when the ‘perm_flag’ is set to 0 and we want to set a particular associative test, the input variable ‘number_rand_perm’ must be inserted in the command line, but any value can be written because it is never used.
2) when ‘dominant’ and ‘recessive’ association tests are computed, for any marker, the terms refer to the effect of B allele over A allele, regardless of which is the minor allele; i.e. the B allele is considered as the minor one.
3) when ‘allelic’, ‘dominant’ and ‘recessive’ test are computed, if the contingency tables contain entries <= 5 then the Fisher exact test is computed.

**Changing individual and marker exclusion criteria**

Inclusion/exclusion of individuals and markers can be obtained by setting appropriately the parameter values in the command line.

For example, let us suppose that we want to specify that individuals with missing genotype more than 0.2 have to be excluded and markers with Minor Allele Frequency less than 0.2 have to be excluded. The command line to use is:

>> compute_association(‘sample_header.txt’, ‘gt.txt’,0,10,’all’,0.2,0.1,0.2)

It is worth noting that even if the threshold value of the marker missing genotype has not to be changed, it must be inserted in the command line.

**Advanced topics and notes**

**Chromosome X processing**

Since the ‘compute_association.m’ program calculates all five test statistics in case-control association test, it is worth noting that, for assessing association of markers on the X chromosome, the following procedures are used. For allelic association test, males are considered as females. For the other four tests, only the female samples are used in analysis, and all males are discarded.

**Parallel computation**

Typically, when one wants to run many permutations, it is possible to run the ‘compute_association.m’ program in parallel in a computational cluster. It is worth noting that each computation has to be run in a different fold to avoid of overwriting of output files.
